# Synthesis, biological investigation, and in silico studies of 2-aminothiazole sulfonamide derivatives as potential antioxidants

**DOI:** 10.17179/excli2024-7855

**Published:** 2025-01-03

**Authors:** Apilak Worachartcheewan, Ratchanok Pingaew, Veda Prachayasittikul, Setthawut Apiraksattayakul, Supaluk Prachayasittikul, Somsak Ruchirawat, Virapong Prachayasittikul

**Affiliations:** 1 Department of Community Medical Technology, Faculty of Medical Technology, Mahidol University, Bangkok 10700, Thailand; 2 Department of Chemistry, Faculty of Science, Srinakharinwirot University, Bangkok 10110, Thailand; 3 Center for Research Innovation and Biomedical Informatics, Faculty of MedicalTechnology, Mahidol University, Bangkok 10700, Thailand; 4 Laboratory of Medicinal Chemistry, Chulabhorn Research Institute, Bangkok 10210, Thailand; 5 Program in Chemical Sciences, Chulabhorn Graduate Institute, Bangkok 10210, Thailand; 6 Center of Excellence on Environmental Health and Toxicology (EHT), Commission on Higher Education, Ministry of Education, Bangkok 10400, Thailand; 7 Department of Clinical Microbiology and Applied Technology, Faculty of MedicalTechnology, Mahidol University, Bangkok 10700, Thailand

**Keywords:** thiazole, sulfonamide, antioxidant, QSAR, computer-aided drug design

## Abstract

Antioxidant compounds have gained current interest as potential protective agents for several therapeutic applications. Antimicrobial drug resistance and infectious diseases also still be concerning globally health issues. Accordingly, the discovery of new antioxidative and antimicrobial agents is essential for human well-being. Thiazole and sulfonamide are privileged scaffolds in drug discovery due to their various bioactive properties. In this study, a series of 2-aminothiazole sulfonamide derivatives (**1-12**) were synthesized and investigated for their antioxidant (i.e., DPPH and SOD-mimic) and antimicrobial activities. Among tested compounds, compound **8 **was the most promising one with potent DPPH and SOD (%DPPH = 90.09 %, %SOD = 99.02 %). However, none of these compounds are active antimicrobial agents. Quantitative structure-activity relationship (QSAR) modeling was performed in which the key findings were further used to guide the rational design of additional derivatives. Two antioxidant QSAR models (i.e., DPPH and SOD) were constructed using multiple linear regression (MLR) with good predictive performance. An additional set of structurally modified compounds were designed based on QSAR findings to finally obtain 112 newly designed compounds in which their activities (DPPH and SOD) were predicted. Most of the modified compounds performed better activities than their prototypes. Mass, polarizability, electronegativity, the presence of C-F bond, van der Waals volume, and structural symmetry were revealed as key properties influencing antioxidant activities. In summary, this study demonstrated the combination used of chemical synthesis, experimental assays, and computer-aided drug design for developing novel antioxidants for potential medicinal applications.

See also the graphical abstract(Fig. 1).

## Introduction

Free radicals are highly reactive molecules containing an unpaired electron (Martemucci et al., 2022[31]). Free radicals are endogenously produced by several physiological processes and intracellular pathways (Chaudhary et al., 2023[10]). Free radicals are harmful molecules capable of destroying cellular components (i.e., DNA, proteins and lipids). These radicals are normally neutralized by the endogenous antioxidant system to maintain oxidant-antioxidant balance and protect against cellular damages (Chaudhary et al., 2023[10]; Martemucci et al., 2022[31]). Oxidative stress is a condition by which the free radicals are excessively accumulated due to the loss of oxidant-antioxidant balance. Oxidative stress is well-recognized to play roles in pathogenesis and progression of many chronic and age-related diseases such as cancer, diabetes mellitus, neurodegenerative diseases, and cardiovascular diseases (Chaudhary et al., 2023[10]). Accordingly, antioxidant molecules are gaining attention as potential protective agents for several oxidative-related conditions. Besides chronic diseases, infectious diseases and antimicrobial drug resistance are considered one of the most concerning public health issues worldwide (Chen et al., 2023[11]; Salam et al., 2023[45]; Urban-Chmiel et al., 2022[49]). The emergence of resistant microbes leads to the ineffectiveness of clinically available antimicrobial drugs. Therefore, the discovery of novel antioxidants and effective antimicrobial agents is essential. 

Natural products are valuable sources of bioactive compounds and privileged scaffolds. Natural-derived pharmacophores are considered as attractive prototypes for effective design of bioactive compounds with therapeutic properties (Nantasenamat and Prachayasittikul, 2015[33]; Prachayasittikul et al., 2015[42]; Roy and Ojha, 2010[43]). Thiazole is a five membered heterocyclic ring containing nitrogen and sulfur atoms (Ayati et al., 2015[3]; Das et al., 2016[15]) found in natural products such as vitamin B1 (thiamine), urukthapelstatin A, and neobacillamide A (Ayati et al., 2015[3]; Davyt and Serra, 2010[16]). Thiazole-based compounds exhibited various pharmacological activities (i.e., antioxidant, antimicrobial, anticancer and antiviral properties) rendering a thiazole scaffold, an attractive pharmacophore in drug design and development (Ayati et al., 2015[3]; Das et al., 2016[15]). Particularly, 2-aminothiazole (2-AT) is the one in the drug discovery spotlight (Ayati et al., 2015[3]; Das et al., 2016[15]). The 2-AT derivatives displayed a variety of biological activities such as antimicrobial, anticancer and anti-inflammatory activities (Das et al., 2016[15]). This core structure is found in many available drugs (i.e., famotidine, cefdinir, abafungin and sudoxican) as well as incorporated in many newly discovered bioactive compounds (Ayati et al., 2015[3]).

Sulfonamide (SO_2_NH) is an important functional moiety in drug design. Sulfonamide derivatives were reported for their antimicrobial, antioxidant, and anticancer activities (Badgujar et al., 2018[5]; Doungsoongnuen et al., 2011[19]; Leechaisit et al., 2019[24]; Sköld, 2000[47]). Particularly, sulfonamides are promising candidates for discovery of antimicrobial agents. Sulfa drugs (i.e., sulfamethoxazole and sulfathiazole) inhibit microbial growth via acting as competitive inhibitors of microbial enzymes involved in folate biosynthetic (Sköld, 2000[47]). Therefore, the sulfonamide moiety is a promising functional group in the discovery of novel antimicrobial as well as other therapeutic agents (Zafar et al., 2023[55]).

Computer-aided drug design has been widely used to facilitate drug development. Understanding structure-activity relationships is essential for successful drug design. Among other tools, quantitative structure‐activity relationship (QSAR) modeling is well-known to effectively reveal the relationship between chemical structures and biological activities of the compounds (Nantasenamat and Prachayasittikul, 2015[33]; Prachayasittikul et al., 2015[42]). The QSAR modeling not only provides a predictive model, but also effectively elucidates key structural properties that are essential for guiding the design of new compounds with preferable properties (Bennani et al., 2022[8]; Prachayasittikul et al., 2015[41]; Worachartcheewan et al., 2020[54]; Zhang et al., 2024[56]). 

This study demonstrates the combination use of chemical synthesis, *in vitro* experiments, and *in silico* tool in discovery of thiazole-based potential antioxidant and antimicrobial agents. A series of 2-aminothiazole sulfonamide derivatives (**1-12**) were synthesized and experimentally investigated for their antioxidant and antimicrobial effects. The experimental results along with the chemical structures of the studied compounds were further used as a data set for QSAR modeling. The constructed models were further applied to guide the design and predict activities of the new derivatives. 

## Materials and Methods

### Chemistry 

The analytical thin-layer chromatography (TLC) was investigated on silica gel 60 F_254_ aluminum sheets. Column chromatography was performed using silica gel 60 (70-230 mesh ASTM). Melting points (mp) were determined using Griffin melting point apparatus and were uncorrected. ^1^H and ^13^C nuclear magnetic resonance (NMR) spectra were recorded on Bruker AVANCE 300 NMR spectrometer or Bruker AVANCE NEO 500 NMR spectrometer. Coupling constants (*J*) were reported in hertz (Hz). The multiplicity of detected signals is expressed as: s (singlet), d (doublet), t (triplet), q (quartet), dd (doublet of doublet), and m (multiplet). High resolution mass spectra (HRMS) were obtained on a Bruker Daltonics (microTOF) mass spectrometer. Reagents for assays included DPPH (2,2-diphenyl-1-picrylhydrazyl), superoxide dismutase (SOD) from bovine erythrocytes, HEPES (*N*-2-hydroxyethylpiperazine-*N*′-2-ethanesulfonic acid), vitamin E, nitro blue tetrazolium (NBT) salt, L-methionine, riboflavin, Triton-100, ciprofloxacin and tetracyclin from Sigma, USA; dimethyl sulfoxide (DMSO), methanol and sodium chloride from Merck, Germany; Mueller Hinton broth (MHB) and Mueller Hinton agar (MHA) from Becton Dickinson, USA. Solvents were analytical grades.

### General procedure for synthesis of thiazole-sulfonamide derivatives 1-12

**Figure 2:**
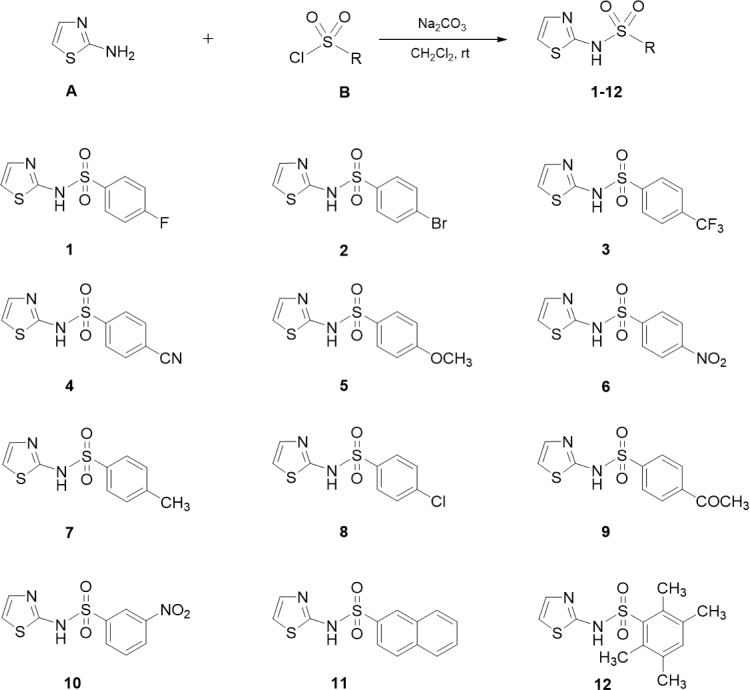
Synthesis of 2-aminothiazole sulfonamides 1-12

A mixture of 2-aminothiazole **A** (2.0 mmol), appropriate sulfonyl chloride **B** (2.0 mmol), and sodium carbonate (3.0 mmol) in dichloromethane (10 mL) was stirred at room temperature (rt) until completion of reaction (monitored by TLC) as shown in Figure 2(Fig. 2). Distilled water (20 mL) was added, and the mixture was extracted with dichloromethane (3 × 30 mL). The organic layer was dried over anhydrous sodium sulfate, filtered and evaporated under reduced pressure. The crude product was further purified by recrystallization or column chromatography on silica gel to obtain pure compound. 

### 4-fluoro-N-(thiazol-2-yl)benzenesulfonamide (1)

From 2-aminothiazole and 4-fluorobenzenesulfonyl chloride. The spectral data have been reported in the literature (Ayimbila et al., 2024[4]).

### 4-bromo-N-(thiazol-2-yl)benzenesulfonamide (2) 

From 2-aminothiazole and 4-bromobenzenesulfonyl chloride. Off-white solid. 35 % yield; mp 214-215 °C. ^1^H NMR (500 MHz, DMSO-*d*_6_) 6.86 (d, *J* = 4.6 Hz, 1H, Ar*H*), 7.28 (d, *J* = 4.6 Hz, 1H, Ar*H*), 7.72 (d, *J* = 8.7 Hz, 2H, Ar*H*), 7.75 (d, *J* = 8.7 Hz, 2H, Ar*H*), 12.84 (s, 1H, N*H*). ^13^C NMR (125 MHz, DMSO-*d*_6_) *δ *108.6, 124.6, 125.7, 127.8, 132.0, 141.6, 169.1. HRMS-TOF: [M+H]^+^ 318.9199 (Calcd for C_9_H_8_BrN_2_O_2_S_2_: 318.9205).

### N-(thiazol-2-yl)-4-(trifluoromethyl)benzenesulfonamide (3) 

From 2-aminothiazole and 4-(trifluoromethyl)benzenesulfonyl chloride. Off-white solid. 44 % yield; mp 199-200 °C. ^1^H NMR (300 MHz, DMSO-*d*_6_) 6.88 (d, *J* = 4.6 Hz, 1H, Ar*H*), 7.29 (d, *J* = 4.6 Hz, 1H, Ar*H*), 7.92 (d, *J* = 8.4 Hz, 2H, Ar*H*), 8.01 (d, *J* = 8.2 Hz, 2H, Ar*H*), 12.94 (s, 1H, N*H*). ^13^C NMR (75 MHz, DMSO-*d*_6_) *δ *108.9, 123.6 (q, ^1^*J_CF_* = 271 Hz), 124.7, 126.3 (q, ^3^*J_CF_* = 4 Hz), 126.7, 131.8 (q, ^2^*J_CF_* = 32 Hz), 146.2, 169.3. HRMS-TOF: [M+Na]^+^ 330.9789 (Calcd for C_10_H_7_F_3_N_2_NaO_2_S_2_: 330.9793).

### 4-cyano-N-(thiazol-2-yl)benzenesulfonamide (4) 

From 2-aminothiazole and 4-cyanobenzenesulfonyl chloride. Dark brown solid. 55 % yield; mp 205-206 °C. ^1^H NMR (300 MHz, DMSO-*d*_6_) 6.90 (d, *J* = 4.6 Hz, 1H, Ar*H*), 7.30 (d, *J* = 4.6 Hz, 1H, Ar*H*), 7.95 (d, *J* = 8.6 Hz, 2H, Ar*H*), 8.02 (d, *J* = 8.5 Hz, 2H, Ar*H*), 12.96 (s, 1H, N*H*). ^13^C NMR (75 MHz, DMSO-*d*_6_) *δ *109.0, 114.5, 117.9, 124.8, 126.5, 133.3, 146.3, 169.3. HRMS-TOF: [M+H]^+^ 266.0054 (Calcd for C_10_H_8_N_3_O_2_S_2_: 266.0052).

### 4-methoxy-N-(thiazol-2-yl)benzenesulfonamide (5) 

From 2-aminothiazole and 4-methyoxybenzenesulfonyl chloride. Off-white solid. 34 % yield; mp 200-201 °C. ^1^H NMR (500 MHz, DMSO-*d*_6_) 3.80 (s, 3H, OC*H_3_*), 6.80 (d, *J* = 4.6 Hz, 1H, Ar*H*), 7.05 (d, *J* = 9.0 Hz, 2H, Ar*H*), 7.23 (d, *J* = 4.6 Hz, 1H, Ar*H*), 7.72 (d, *J* = 8.9 Hz, 2H, Ar*H*), 12.64 (brs, 1H, N*H*). ^13^C NMR (125 MHz, DMSO-*d*_6_) *δ *55.6, 108.0, 114.1, 124.4, 127.9, 134.2, 161.9, 168.7. HRMS-TOF: [M+H]^+^ 271.0206 (Calcd for C_10_H_11_N_3_O_3_S_2_: 271.0206).

### 4-nitro-N-(thiazol-2-yl)benzenesulfonamide (6) 

From 2-aminothiazole and 4-nitrobenzenesulfonyl chloride. Off-white solid. 43 % yield; mp 265-266 °C. ^1^H NMR (500 MHz, DMSO-*d*_6_) 6.91 (d, *J* = 4.6 Hz, 1H, Ar*H*), 7.31 (d, *J* = 4.6 Hz, 1H, Ar*H*), 8.04 (d, *J* = 8.9 Hz, 2H, Ar*H*), 8.36 (d, *J* = 8.9 Hz, 2H, Ar*H*), 13.00 (s, 1H, N*H*). ^13^C NMR (125 MHz, DMSO-*d*_6_) *δ *109.1, 124.5, 124.9, 127.3, 147.8, 149.3, 169.3. HRMS-TOF: [M+H]^+^ 285.9959 (Calcd for C_9_H_8_N_3_O_4_S_2_: 285.9951). The spectroscopic data are in accordance with the reported literature (Dea-Ayuela et al., 2009[17]).

### 4-methyl-N-(thiazol-2-yl)benzenesulfonamide (7) 

From 2-aminothiazole and 4-toluenesulfonyl chloride. Light brown solid. 69 % yield; mp 210-212 °C. ^1^H NMR (300 MHz, DMSO-*d*_6_) 2.34 (s, 3H, C*H_3_*), 6.81 (d, *J* = 4.6 Hz, 1H, Ar*H*), 7.24 (d, *J* = 4.6 Hz, 1H, Ar*H*), 7.33 (d, *J* = 8.1 Hz, 2H, Ar*H*), 7.68 (d, *J* = 8.6 Hz, 2H, Ar*H*), 12.70 (s, 1H, N*H*). ^13^C NMR (75 MHz, DMSO-*d*_6_) *δ *20.7, 107.8, 124.1, 125.6, 129.0, 139.5, 141.8, 168.5. HRMS-TOF: [M+H]^+^ 255.0260 (Calcd for C_10_H_11_N_2_O_2_S_2_: 255.0256). The spectroscopic data are in accordance with the reported literature (Lu et al., 2018[27]).

### 4-chloro-N-(thiazol-2-yl)benzenesulfonamide (8) 

From 2-aminothiazole and 4-chlorobenzenesulfonyl chloride. Off-white solid. 35 % yield; mp 203-205 °C. ^1^H NMR (500 MHz, DMSO-*d*_6_) 6.85 (d, *J* = 4.6 Hz, 1H, Ar*H*), 7.27 (d, *J* = 4.6 Hz, 1H, Ar*H*), 7.60 (d, *J* = 8.7 Hz, 2H, Ar*H*), 7.79 (d, *J* = 8.7 Hz, 2H, Ar*H*), 12.84 (brs, 1H, N*H*). ^13^C NMR (125 MHz, DMSO-*d*_6_) *δ *108.5, 124.6, 127.7, 129.1, 136.7, 141.2, 169.0. HRMS-TOF: [M+H]^+^ 274.9712 (Calcd for C_9_H_8_ClN_2_O_2_S_2_: 274.9710). The spectroscopic data are in accordance with the reported literature (Dea-Ayuela et al., 2009[17]).

### 4-acetyl-N-(thiazol-2-yl)benzenesulfonamide (9)

From 2-aminothiazole and 4-acetylbenzenesulfonyl chloride. Brown solid. 34 % yield; mp 207-208 °C. ^1^H NMR (300 MHz, DMSO-*d*_6_) 2.60 (s, 3H, COC*H_3_*), 6.86 (d, *J* = 4.6 Hz, 1H, Ar*H*), 7.27 (d, *J* = 4.6 Hz, 1H, Ar*H*), 7.92 (d, *J* = 8.3 Hz, 2H, Ar*H*), 8.08 (d, *J* = 8.4 Hz, 2H, Ar*H*), 12.87 (s, 1H, N*H*). ^13^C NMR (75 MHz, DMSO-*d*_6_) *δ *26.7, 108.4, 124.3, 125.9, 128.6, 139.2, 146.0, 169.0, 197.1. HRMS-TOF: [M+H]^+^ 283.0209 (Calcd for C_11_H_11_N_2_O_3_S_2_: 283.0206).

### 3-nitro-N-(thiazol-2-yl)benzenesulfonamide (10) 

From 2-aminothiazole and 3-nitrobenzenesulfonyl chloride. Off-white solid. 47 % yield; mp 204-205 °C. ^1^H NMR (300 MHz, DMSO-*d*_6_) 6.91 (d, *J* = 4.6 Hz, 1H, Ar*H*), 7.31 (d, *J* = 4.6 Hz, 1H, Ar*H*), 7.85 (t, *J* = 8.0 Hz, 1H, Ar*H*), 8.22 (d, *J* = 7.8 Hz, 1H, Ar*H*), 8.43 (dd, *J* = 8.1, 2.2 Hz, 1H, Ar*H*), 8.47 (d, *J* = 1.7 Hz, 1H, Ar*H*) 13.00 (s, 1H, N*H*). ^13^C NMR (75 MHz, DMSO-*d*_6_) *δ *109.0, 120.2, 124.8, 126.6, 131.1, 131.7, 143.9, 147.7, 169.3. HRMS-TOF: [M+H]^+^ 307.9770 (Calcd for C_9_H_7_N_3_NaO_4_S_2_: 307.9770).

### N-(thiazol-2-yl)naphthalene-2-sulfonamide (11) 

From 2-aminothiazole and 2-naphthalenesulfonyl chloride. Brown solid. 36 % yield; mp 206-207 °C. ^1^H NMR (300 MHz, DMSO-*d*_6_) 6.83 (d, *J* = 4.6 Hz, 1H, Ar*H*), 7.26 (d, *J* = 4.6 Hz, 1H, Ar*H*), 7.60-7.70 (m, 2H, Ar*H*), 7.80 (dd, *J* = 8.6, 1.8 Hz, 1H, Ar*H*), 8.01 (dd, *J* = 6.9, 2.1 Hz, 1H, Ar*H*), 8.07 (d, *J* = 8.7 Hz, 1H, Ar*H*), 8.15 (dd, *J* = 6.9, 2.2 Hz, 1H, Ar*H*), 8.46 (s, 1H, Ar*H*), 12.79 (s, 1H, N*H*). ^13^C NMR (75 MHz, DMSO-*d*_6_) *δ *108.0, 122.0, 124.2, 125.8, 127.2, 127.5, 128.1, 128.8, 131.5, 133.8, 139.3, 168.7. HRMS-TOF: [M+H]^+^ 313.0075 (Calcd for C_13_H_10_N_2_NaO_2_S_2_: 313.0076).

### 2,3,5,6-tetramethyl-N-(thiazol-2-yl)benzenesulfonamide (12) 

From 2-aminothiazole and 2,3,5,6-tetramethylbenzenesulfonyl chloride. Dark brown solid. 68 % yield; mp 201-202 °C. ^1^H NMR (300 MHz, DMSO-*d*_6_) 2.20 (s, 6H, 2 × C*H_3_)_, _*2.50 (s, 6H, 2 × C*H_3_)_, _*6.75 (d, *J* = 4.6 Hz, 1H, Ar*H*), 7.15 (s, 1H, Ar*H*), 7.22 (d, *J* = 4.6 Hz, 1H, Ar*H*), 12.53 (s, 1H, N*H*). ^13^C NMR (75 MHz, DMSO-*d*_6_) *δ *17.8, 20.5, 107.8, 124.3, 134.0, 134.3, 135.1, 140.3, 167.8. HRMS-TOF: [M+H]^+^ 297.0723 (Calcd for C_13_H_17_N_2_O_2_S_2_: 297.2726).

### Biological activities 

#### Antioxidant activity assays

Antioxidant activities of thiazole derivatives (**1**-**12**) were evaluated for assessing the ability of the compounds to scavenge or neutralize the free radicals and superoxide anion using 1,1-diphenyl-2-picrylhydrazyl (DPPH) and superoxide dismutase (SOD) assays, respectively.

The radical scavenging (DPPH) activity was investigated using 1,1-diphenyl-2-picrylhydrazyl (DPPH) assay (Worachartcheewan et al., 2022[53]). The DPPH is a stable purple colored compound used to represent free radicals. The color of testing solution was changed upon the reaction with antioxidant compound, in which the hydrogen atom or electron was donated to the DPPH radical to produce a light-yellow colored product (1,1-diphenyl-2-picrylhydrazine) (Hussen and Endalew, 2023[22]). The 0.1 mM DPPH solution was prepared in methanol and stored at 4 °C. The test tubes containing 0.45 mL of the tested compound solution (dissolved in DMSO solvent) were prepared. The assay was initiated by adding 1 mL of 0.1 mM methanolic DPPH solution in each test to give a final concentration of 300 μg/mL. The reaction was mixed and incubated in a dark condition for 30 min. After that, the absorbance was measured at 517 nm using UV-Visible spectrophotometer (UV-1610, Shimadzu Corporation, Japan). The percentage of radical scavenging activity (RSA) or %DPPH was calculated according to equation (1).

DPPH (%) = (1 - *Abs*._sample_ / *Abs*._control_) × 100 (1)

where *Abs*._control_ is the absorbance of the control reaction without the tested compounds, and *Abs.*_sample_ is the absorbance of tested compound. α-Tocopherol was used as a control experimentation and methanol was used as a blank reaction. All determinations were performed in triplicate.

The superoxide dismutase (SOD) activity was determined using SOD assay (Piacham et al., 2003[38]). The assay was performed by mixing 1 mL of stock solution (containing 27 mL of HEPES buffer (50 mM, pH 7.8), 1.5 mL of L-methionine (30 mg/mL), 1 mL of nitro blue tetrazolium (NBT, 1.41 mg/mL) and 0.75 mL of Triton X-100 (1 wt %)) in each test tube containing 0.45 mL solution of the tested compound (dissolved in DMSO solvent) to yield a final concentration of 300 µg/mL. The reaction was initiated by adding 10 μL of riboflavin (44 μg/mL) and then was excited under a Philips Classic Tone lamp (60 W) in a light box for 7 min. The superoxide anions were generated in the mixture reaction by riboflavin under the light. The excited riboflavin receives donated electron from methionine to produce a semiquinone. The oxygen (O_2_) of the superoxide anion receives electrons from the semiquinone and reacts with NBT to form a purple formazan product. The color of solution was changed to purple due to the NBT photoreduction. If the tested compounds possess antioxidant effect, the superoxide anions were scavenged, and the solution remained in light-yellow color. The absorbance of NBT photoreduction was measured at 550 nm using UV-Vis spectrophotometer (UV-1610, Shimadzu Corporation, Japan). The percentage of inhibition as SOD activity was computed using equation (2).

SOD (%) = (1 - *Abs*._sample_ / *Abs*._control_) × 100 (2)

where *Abs*._control_ is the absorbance of the control reaction without the tested compounds, and *Abs.*_sample_ is the absorbance of the tested compound. Superoxide dismutase (SOD) from bovine erythrocytes was used as a control experimentation. All determinations were performed in triplicate.

All compounds were initially screened using the highest concentration at 300 µg/mL and the %DPPH or %SOD were calculated. Compounds exhibiting %DPPH or %SOD greater than 50 % were further investigated using serial dilutions to determine their IC_50_ values (a concentration required for inhibiting 50 % of radicals). The IC_50_ was finally calculated by plotting the measured absorbance values versus the tested diluted concentrations. 

#### Antimicrobial activity assay

The antimicrobial activity of the studied compounds was determined using conventional agar dilution method (Baron et al., 1994[7]; Cherdtrakulkiat et al., 2020[12]). The antibacterial drugs included ciprofloxacin and tetracycline were employed as control drugs. The MIC quality control (QC) ranges in μg/mL of the antimicrobial drugs was recommended by Clinical & Laboratory Standards Institute (CLSI, 2013[13]). The tested compounds and reference antibacterial drugs (i.e., ciprofloxacin and tetracycline as controls of the testing system) were individually dissolved in DMSO solvent. The serial dilution was performed using Mueller Hinton broth (MHB) to prepare the concentrated solution of the compounds. Then, 1 mL of prepared compound solutions were transferred into test tubes containing 19 mL of Mueller Hinton agar (MHA) to give the final concentrations of 4-256 μg/mL. Finally, agar plates containing diverse concentrations of tested compounds or control agents were prepared.

A variety of microorganisms (i.e., gram-positive and gram-negative bacteria) as well as diploid fungus (yeast) were used for antimicrobial study. Twenty-nine strains of microorganisms comprised of reference strains and clinical isolates were used for antimicrobial activity testing included **gram positive bacteria:** *Staphylococcus aureus* ATCC 29213, *Staphylococcus aureus* ATCC 25923, methicilin resistant *staphylococcus aureus* JCSC 3063, methicilin resistant *staphylococcus aureus* N315, methicilin resistant *Staphylococcus aureus* JCSC 4788, *Staphylococcus epidermidis* ATCC 12228, *Enterococcus faecalis* ATCC 29212, *Enterococcus faecalis* ATCC 33186, *Micrococcus luteus ATCC* 10240, *Corynebacterium diphtheriae* NCTC 10356, *Bacillus subtilis* ATCC 6633, *Bacillus cereus*,* Listeria monocytogenes*; **gram negative bacteria:** *Escherichia coli* ATCC 25922, *Klebsiella pneumoniae* ATCC 700603, *Serratia macescens* ATCC 8100, *Salmonella typhimurium* ATCC 13311, *Shewanella putrefaciens* ATCC 8071, *Achromobacter xylosoxidans* ATCC 2706, *Pseudomonas aeruginosa* ATCC 27853, *Pseudomonas stutzeri* ATCC 17587, *Shigella dysenteriae*, *Salmonella enteritidis*, *Morganella morganii*, *Aeromonas hydrophila*, *Citrobacter freundii*, *Plesiomonas shigelloides* and **diploid fungus (yeast):** *Candida albicans* ATCC 90028 and *Saccharomyces cerevisiae* ATCC 2601.

The microbes were cultured in MHB at 37 °C for 24 h and were diluted with 0.9 % normal saline solution (NSS) for adjusting the microorganism cell density to give 1×10^8^ CFU/mL (which is equivalent to 0.5 McFarland standards). The suspensions were then further diluted with 0.9 % NSS to obtain a final microorganism cell density of 1×10^7^ CFU/mL. The microorganisms were inoculated onto each plate containing various concentrations of tested compound using amultipoint inoculator (Denley-Teck Ltd., England) and further incubated at 37°C for 24-48 h. In addition, the MHB and DMSO solvent without tested compounds and antibacterial drugs (i.e., ciprofloxacin and tetracyclin) were used as the controls to ensure that the solvents having none of antimicrobial effect or the testing system is not contaminated. Finally, the minimum inhibitory concentration (MIC), which is the lowest concentration of the compounds to inhibit the growth of microorganisms, was determined.

### QSAR study

**Figure 3:**
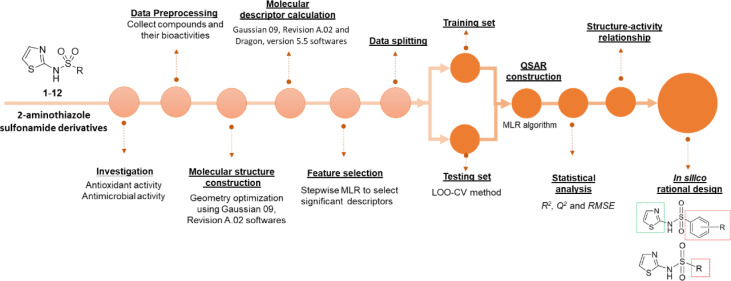
Schematic workflow of QSAR modeling and QSAR-guided rational design

Descriptors are numerical variables representing properties of compounds. The descriptor values were obtained from structural calculations and were further used as predictors (X variables), whereas the bioactivity values were utilized as dependent variable (Y variable) for QSAR modeling. A schematic workflow of constructing QSAR model and *in silico* rational design are presented in Figure 3(Fig. 3).

### Data pre-processing

The experimentally obtained bioactivity values (i.e., antioxidant (% or IC_50_) and antimicrobial (MIC) values) along with chemical structures of the studied compounds (**1-12**) were used to prepare data sets for QSAR modeling. Inactive compounds were excluded from the data sets. Antimicrobial activity values expressed as MIC were prior converted to pMIC (-logMIC) by taking negative log10 to normalize the data points, while the same pre-processing is not required for the antioxidant activity expressed as %DPPH pr %SOD.

### Chemical structure construction and descriptors calculation

Chemical structures of the compounds were drawn using GaussView, version 3.09 (Dennington et al., 2003[18]), and were geometrically optimized using Gaussian 09, Revision A.02 at the semi-empirical level using Austin Model 1 (AM1), followed by the density functional theory (DFT) calculation using Becke's three-parameter hybrid method and the Lee-Yang-Parr correlation functional (B3LYP) together with the 6-31 g(d) basis set (Frisch et al., 2009[20]). The optimized structures were used as input files for extracting a set of 13 quantum chemical descriptors (i.e., the total energy (E_total_), the highest occupied molecular orbital energy (E_HOMO_), the lowest unoccupied molecular orbital energy (E_LUMO_), the total dipole moment (µ) of the molecule, the electron affinity (EA), the ionization potential (IP), the energy difference of HOMO and LUMO (HOMO-LUMO_Gap_), Mulliken electronegativity (χ), hardness (η), softness (S), electrophilicity (ω), electrophilic index (ω_i_) and the mean absolute atomic charge (Q_m_) (Pingaew et al., 2022[40]). Moreover, the optimized structures were further used for calculation of an additional set of 3,224 molecular descriptors, which were calculated using Dragon software, version 5.5 (Talete srl., 2007[48]). The calculated molecular descriptors included 22 categories: 48 constitutional descriptors, 119 topological descriptors, 47 Feature selection of descriptors walk and path counts, 33 connectivity indices, 47 information indices, 96 2D autocorrelation, 107 edge adjacency indices, 64 burden eigenvalues, 21 topological charge indices, 44 eigenvalue-based indices, 41 randic molecular profiles, 74 geometrical descriptors, 150 RDF descripttors, 160 3D-MoRSE descriptors, 99 WHIM descriptors, 197 GETAWAY descriptors, 154 functional group counts, 120 atom-centred fragments, 14 charge descriptors, 29 molecular properties, 780 2D binary fingerprints and 780 2D frequency fingerprints.

### Descriptors selection

Descriptors selection was performed to select a final set of important descriptors for QSAR modeling. The calculated set of 3,224 molecular descriptors was initially filtered by excluding the one with constant value. A pair correlation between descriptors in this set was calculated to determine multi-collinearity, and the pair-descriptors with correlation coefficient >0.99 were excluded. Subsequently, the remaining set of molecular descriptors was combined with 13 quantum chemical descriptors, and was subjected to further selection to select a final set of important descriptors by stepwise MLR using SPSS statistics 18.0, SPSS Inc., USA) or by automatic variable selection procedure (CfsSubsetEval combined with the BestFirst) using Waikato Environment for Knowledge Analysis (Weka), version 3.4.5 (Witten et al., 2011[51]). Furthermore, the intercorrelation matrix between each pair of selected descriptors was calculated by Pearson's correlation coefficient (r) using SPSS statistics 18.0 (SPSS Inc., USA) to ensure their independence. Any pairs of descriptors with |r| ≥ 0.9 was defined as highly correlated predictors, and one of them was excluded (Nowaczyk and Kulig, 2012[35]).

### Data splitting

The data set was divided into 2 subsets including training and leave-one-out cross-validation (LOO-CV) sets. The training set was used to train and generate machine learning-based QSAR model, whereas the LOO-CV set was employed to validate the models. To validate the performance of the model, one sample was excluded from the whole data set to be used as a testing set, while the remaining N−1 sample was used as the training set. This sampling process was repeated iteratively until every sample in the data set was used as the testing set (Leechaisit et al., 2019[24]).

### Multivariate analysis

Multiple linear regression (MLR) is a machine learning algorithm to generate a linear interpretable mathematical equation which correlates between significant descriptors (X variables, independent variables) and biological activities (Y, dependent variables). The QSAR models were constructed using multiple linear regression (MLR) according to equation (3)

*Y* = *B*_0_ + Σ *B*_n_*X*_n_ (3)

where *Y* is the biological activity, *B_0_* is the intercept, and *B_n_* are the regression coefficients of the descriptors *X_n_*. The MLR method was performed using Weka, version 3.4.5 (Witten et al., 2011[51]).

### Model evaluation

Statistical parameters (i.e., squared correlation coefficient (*R^2^_Tr _*for training set), predictivity (*Q^2^_LOO-CV_* for testing set), and root mean squared error (*RMSE_Tr_* and* RMSE_LOO-CV_* of training and LOO-CV sets, respectively) were calculated to assess the performance of the constructed models (Nantasenamat et al., 2010[32]). Good predictive performance is indicated for the model providing high values of *R^2^_Tr_* and *Q^2^_LOO-CV_*, but low *RMSE* values. 

### In silico guided rational design

Original compounds exhibiting antioxidant activities (**1-5**, and** 7-11**) were selected as templates of design of new derivatives. Structural modification on the core of templates was performed according to key descriptors presented in the QSAR models. A variety of chemical functional groups (R) were substituted or introduced to the templates. Chemical structures of the newly designed compounds were constructed using GaussView, version 3.09 (Dennington et al., 2003[18]). Geometrical optimization and descriptor calculation were performed in the same manner with the original compounds as mentioned above. Values of key selected descriptors presented in the QSAR equations then were prepared for predicting activities of the modified compounds using the constructed models (Prachayasittikul et al., 2015[41]; Worachartcheewan et al., 2020[54]). The newly designed compounds displaying higher activity than the prototypes were summarized as potential compounds for further development.

.

## Results and Discussion

### Synthesis of 2-aminothiazole sulfonamide derivatives

Twelve 2-aminothiazole sulfonamides (**1-12**) were synthesized by *N*-sulfonylation of 2-aminothiazole **A** with the corresponding benzenesulfonyl chlorides **B** in the presence of sodium carbonate in dichloromethane at room temperature as shown in Figure 2(Fig. 2). Structures of the 2-aminothiazole sulfonamides (**1-12**) were confirmed based on their ^1^H and ^13^C NMR and HRMS data. In ^1^H NMR spectra, the two aromatic protons of thiazole ring were assigned at *δ *in the range of 6.75-6.91 ppm and 7.22-7.31 ppm as two doublets with coupling constant of *J *= 4.6 Hz, whereas protons of the aromatic ring attached to the sulfonyl moiety showed signals in the region between 7.05-8.47 ppm. In addition, all synthesized sulfonamides had molecular ion peaks corresponding to their molecular formula.

### Biological activities

#### DPPH activity 

All studied compounds (**1-12**) were initially tested with the highest concentration at 300 μg/mL as shown in Table 1(Tab. 1). It was found that all tested compounds displayed DPPH activity providing %DPPH = 7.58 - 90.09 %. Interestingly, compound **8** containing chloride group at *para*-position on benzene ring displayed the highest DPPH activity (90.09 %). Five top-ranking compounds were listed as compound **8** (*p*-Cl, 90.09 %) >**10 **(*m*-NO_2_, 70.29 %) >**12** (2,3,5,6-tetraCH_3_, 41.97 %) >**6** (*p*-NO_2_, 33.96 %) >**5** (*p*-OCH_3_, 33.33 %). In overview, most of the synthesized compounds provided improved radical scavenging activities when comparing to the prototype 2-AT (%DPPH = 11.03 %). Among these, only two compounds (**8** and **10**) displayed %DPPH activity more than 50 % and were further tested to determine their IC_50 _values (i.e., IC_50_: **8** = 109.73 µM, and **10 **= 484.25 µM). However, both compounds provided lesser activity when compared to the reference α-tocopherol (IC_50_ = 8.20 µM). 

#### SOD activity 

SOD-mimic activity of the 2-aminothiazole sulfonamide derivatives (**1-12**) were investigated at 300 μg/mL, Table 1(Tab. 1). It was found that all compounds displayed SOD activity providing %SOD from 5.41 to 99.02 %, except for compound **1** with inactive activity. Like DPPH activity, compound **8** (*p*-Cl) provided the highest antioxidant activity with 99.02 % followed by compound compound **10** (*m*-NO_2_, 92.05 %). Among all, five compounds included compound **6 **(64.14 %), **8 **(99.02 %), **9** (50.54 %), **10 **(92.05 %) and **12** (69.31 %) exhibited %SOD greater than 50 %, and were further serial diluted to determine their IC_50_. The IC_50_ values of compounds **6, 8, 9, 10 **and** 12 **were 591.88 µM, 188.27 µM, 1090.24 µM, 362.67 µM and 606.78 µM, respectively. Top five most potent compounds were ranked as compounds **8** (*p*-Cl) >**10** (*m*-NO_2_) >**6** (*p*-NO_2_) >**12** (2,3,5,6-tetraCH_3_) >**9** (*p*-COCH_3_). The two most potent compounds **8 **and** 10** provided preferable activity (IC_50_: **8** = 188.27 and **10** = 362.67 µM), but these compounds were less potent than the reference bovine SOD (IC_50_ = 0.01 µM). However, it was noted that all top five compounds are more potent than their prototype 2-AT (IC_50_ = 1744.36 µM). 

#### Antimicrobial activity

The agar dilution method was used to determine antimicrobial potential of the compounds (Balouiri et al., 2016[6]). It was found that ciprofloxacin displayed MIC values of 0.5 μg/mL for *S. aureus* ATCC 29213 and *P. aeruginosa* ATCC 27853, and MIC values of 1 μg/mL for *E. faecalis* ATCC 29212 which the MIC QC ranges of the microorganism were 0.12-0.5 μg/mL, 0.12-1 μg/mL and 0.25-2 μg/mL for *S. aureus* ATCC 29213, P*. aeruginosa* ATCC 27853 and *E. faecalis *ATCC 29212, respectively. The tetracycline gave MIC value of 1 μg/mL against *S. aureus* ATCC 29213, MIC value of 2 μg/mL against *E. coli* ATCC25922, and MIC value of 32 μg/mL against *E. faecalis* ATCC 29212 and *P. aeruginosa* ATCC 27853. The tetracycline with MIC QC ranges of the microorganism were 0.12-1 μg/mL, 0.5-2 μg/mL, 8-32 μg/mL and 8-32 μg/mL for *S. aureus* ATCC 29213, *E. coli* ATCC25922, *E. faecalis* ATCC 29212 and *P. aeruginosa* ATCC 27853, respectively. The MIC values of ciprofloxacin and tetracycline were in the MIC QC ranges against bacterial strains' control (reference strains) according to CLSI (2013[13]). In addition, the control (i.e., DMSO solvent and MHB) spots indicated that the solvent itself showed no effect on microbial growth and there is no contamination in the system. Accordingly, the testing system was reliable and acceptable for further testing and interpretating activities of the tested compounds. All twelve compounds (**1-12**) were tested, unfortunately, none of them exhibit antimicrobial activities at concentration range of 4-256 µg/mL. The prototype, 2-AT also did not exhibit antimicrobial property.

#### QSAR models 

QSAR modeling is widely employed to facilitate drug design and discovery (Arthur et al., 2016[1]; Lu et al., 2022[26]; Phanus-Umporn et al., 2020[37]; Pingaew et al., 2021[39]; Verma et al., 2017[50]). The QSAR modeling was performed as a tool for guiding the rational design of several kinds of novel bioactive compounds (Bennani et al., 2022[8]; Prachayasittikul et al., 2015[41]; Worachartcheewan et al., 2020[54]; Zhang et al., 2024[56]). In this study, two QSAR models were constructed according to the presented antioxidant (i.e., DPPH and SOD) activities of the tested compounds. All compounds were inactive antimicrobial agents, therefore, the QSAR study on antimicrobial activity was not performed. Only active compounds were included in the data set. Compound **1** showing inactive SOD activity was removed from the SOD data set. Accordingly, there were 12 and 11 compounds included in the data sets of DPPH and SOD models, respectively. Because only some of the compounds are highly active enough to calculate IC_50_ values, bioactivity value in percentage form (i.e., %DPPH and %SOD) was used for constructing the models. 

The compounds were drawn, geometrically optimized, and calculated to obtain an original set of 3,224 molecular descriptors. The calculated molecular descriptors were initially filtered to remain a set of 1,328 molecular descriptors. These filtered descripttors were combined with 13 calculated quatum chemical descriptors to give a set of 1,341 descriptors which was subjected to final feature selection to select important descriptors for DPPH model (i.e., RDF040m, H6m, B01[C-F] and HATS8p) and SOD model (i.e., Gu, Mor31m, Mor13e and H0v). Definition of these selected descriptors are provided in Table 2(Tab. 2). The intercorrelation between each pair of descriptors were also determined using the cutoff value of |r| ≥ 0.9. It was found that every pair of the selected descriptors displayed |r| < 0.9 indicating that each descriptor was independent without any collinearity (Supplementary information, Tables S1 and S2). Values of selected descriptors of the compounds are provided in Tables 3(Tab. 3) and 4(Tab. 4). 

**Figure 4:**
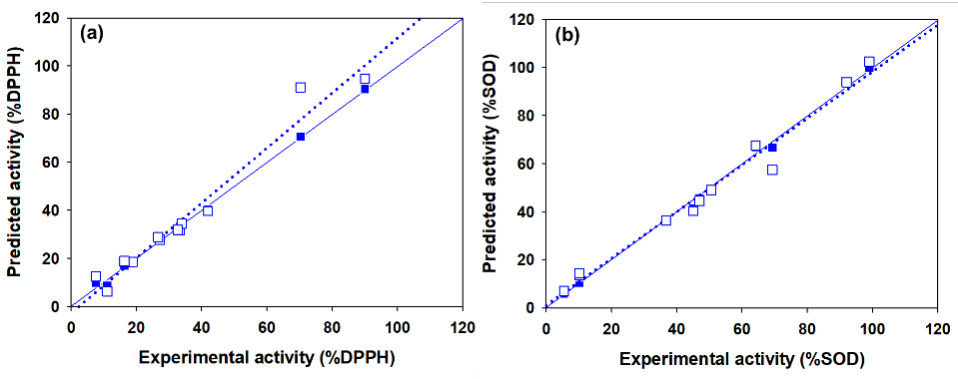
Plot of the experimental versus predicted %DPPH (a) and %SOD (b) activities. The white square symbol and solid regression line are represented as training set whereas blue square symbol and dotted regression line are represented as the leave-one-out cross validation

Two antioxidant QSAR (i.e., DPPH and SOD) models were successfully constructed using MLR method (equation (3)) as shown in Table 5(Tab. 5). Results indicated that two constructed models provided acceptable predictive performance. SOD model provided *R_Tr_*^2^ = 0.9956 and *RMSE_Tr_* = 2.0039 for training set; *Q_LOO-CV_*^2^ = 0.9773 and *RMSE_LOO-CV _* = 4.5756 for LOO-CV set (Table 5(Tab. 5)). DPPH model displayed *R_Tr_*^2^ = 0.9966 and *RMSE_Tr_* = 1.3337 for training set; *Q_LOO-CV_*^2^ = 0.9614 and *RMSE_LOO-CV _* = 6.6143 for LOO-CV set (Table 5(Tab. 5)). Values of experimental versus predicted %DPPH and %SOD are provided in Tables 3(Tab. 3) and 4(Tab. 4), and their scatter plots are shown in Figure 4a and 4b(Fig. 4). The plots indicated that the predicted values were highly correlated with the experimental values.

### In silico guided rational design and structure-activity relationship

The parent 2-AT displayed DPPH and SOD activities with 11.03 % and 65.63 % (IC_50_ = 1744.36 µM), respectively, but demonstrated none of antimicrobial activity. From the findings of tested compounds (**1-12**), both DPPH and SOD activities were improved when the core of 2-AT was linked to the sulfonamide moiety and various functional groups were introduced into the attached benzene ring. However, the modification of the 2-AT by introducing sulfonamide moiety showed no effect on antimicrobial activity although the sulfonamide group has been recognized for the design and synthesis of antimicrobial drugs (Ovung and Bhattacharyya, 2021[36]).

Among original compounds, the chloro-compound **8** (R = Cl at *para*-position on benzene ring) was shown to be the most promising one with the highest DPPH and SOD activities (90.09 % and 99.02 %, respectively) followed by the nitro-compound **10** (R = Cl at *para*-position on benzene ring, %DPPH = 70.29 and %SOD = 92.05). It was noticed that both activities were decreased for the fluoro-compound **1** (DPPH = 7.58 % and inactive SOD).

The DPPH model indicated that mass (RDF040m, H6m), presence/absence of C-F and polarizability (HATS8p) were significant properties for DPPH activity. The positive regression coefficient values of RDF040m and H6m indicated that the increasing values of these descriptors promote higher %DPPH, while the negative regression coefficient values of B01[C-F] and HATS8p suggested their decreasing values are required for preferabley DPPH activity. Among all key descriptors, mass (H6m) of the compounds played major influence on the activity followed by polarizability (HATS8p). Considering the most potent compound **8**, the high %DPPH was mainly due to its highest mass along with absence of C-F bond in the molecule (RDF040m = 10.949, B01[C-F] = 0, Table 3(Tab. 3)). It was noted that the lowest DPPH activity of fluoro-compound 1 was due to the presence of C-F bond (B01[C-F] = 1) as well as its lowest mass descriptor value (H6m = 0.0005). Similar effect of C-F bond was also noted for another fluoro-compound **3** with B01[C-F] = 1 (Table 3(Tab. 3)). In addition, the position of substituted R group on the benzene ring also affected DPPH activity of the compounds. As observed for both nitro-compounds, changing the substituted position from *para*-position (compound **6**) to *meta*-position (compound **10**) notably increased the values of two mass descriptors (i.e., RDF040m and H6m) leading to notable increase of the DPPH activity (**6**: RDF040m = 6.014, H6m = 0.009, %DPPH = 33.96, **10**: RDF040m = 6.161, H6m = 0.143, %DPPH = 70.29) (Table 3(Tab. 3)).

The SOD model indicated that total symmetry index (Gu), mass (Mor31m), Sanderson electronegativity (Mor13e) and van der Waals volume (H0v) are essential properties governing SOD-mimic activity. According to regression coefficient values, low values of Gu and Mor13e, but high values of Mor31m and H0v were required for high %SOD. The most potent SOD activity of compound **8** was noted to be due to its high Mor31m (0.372) and H0v (1.523) values, but low Gu (0.198) and Mor13e (0.205) values. Like the DPPH, better SOD activity was observed for compound **10 **bearing *meta*-NO_2_ when compared to its *para*-NO_2_ derivative **6**. This could be due to the decreased value of total symmetry index (Gu), but increased values of mass (Mor31m) and van der Waal volume (Hov) descriptors (**6**: Gu = 0.196, Mor31m = 0.181, Hov = 1.309, %SOD = 64.14 %, **10**: Gu = 0.188, Mor31m = 0.203, H0v = 1.325, %SOD = 92.05 %) (Table 4(Tab. 4)).

**Figure 5:**
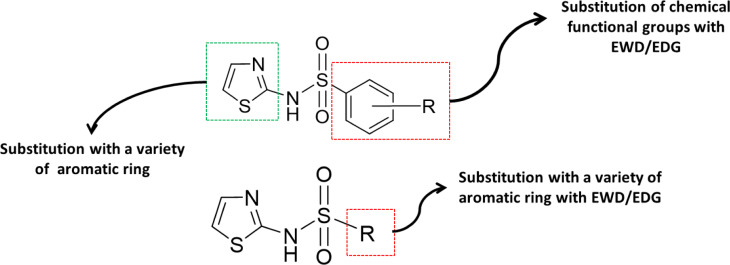
Structural modification strategy to obtain an additional set of 112 modified compounds. Various functional groups (i.e., electron withdrawing groups (EWG), electron donating group (EDG) and aromatic ring) were substituted on the core of the parent templates

Nine original compounds (**1, 2, 3, 4, 5, 7, 9, 10 **and** 11**) with antioxidant (i.e., DPPH and/or SOD) activities were used as templates for the design of new derivatives. Structural modifications on the core of templates were performed according to QSAR findings. Various types of functional groups such as electron-donating groups (EDG), electron-withdrawing groups (EWG), aromatic or heterocyclic rings (i.e., furan, thiophene and pyrrole) were substituted on the core of the prototypes (Figure 5(Fig. 5)). Finally, a total set of 112 modified compounds were designed from prototypes **1, 2, 3, 4, 5, 7, 9, 10 **and** 11** to give 1, 26, 26, 6, 11, 26, 1, 1, and 14 modified compounds, respectively (Supplementary information, Table S3 and Figures S1-S7). The newly designed compounds were drawn, optimized, and calculated to obtain values of key descriptors, which subsequently used to predict their antioxidant activities using the constructed DPPH and SOD models. Values of key descriptors and predicted activities of the modified compounds are provided in Supplementary information, Tables S4 and S5.

**Figure 6:**
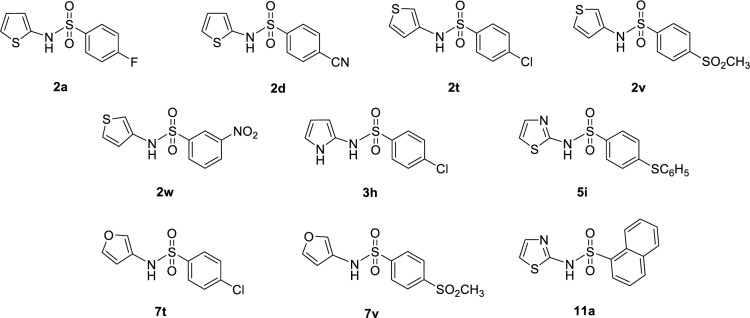
Top ten modified compounds with the highest predicted DPPH activity (%)

**Figure 7:**
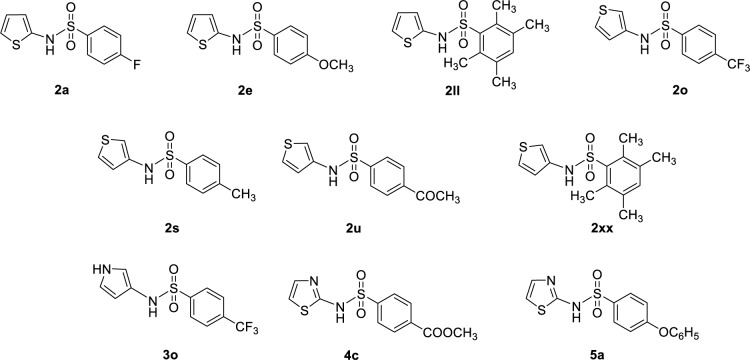
Top ten modified compounds with the highest predicted SOD activity (%)

In overview, the modified compounds displayed improved activities than their prototypes (i.e., 88 compounds for DPPH and 64 compounds for SOD activities, Supplementary information, Table S3). Chemical structures of ten modified compounds with top-ranked DPPH and SOD activities are summarized in Figures 6(Fig. 6) and 7(Fig. 7), and their predicted antioxidant activities are provided in Supplementary information, Table S6.

It was found that most of the newly designed compounds displaying highly predicted DPPH activity are compounds bearing heterocyclic rings (i.e., furan, thiophene and thiazole) on the amino group of sulfonamide core whereas their terminal benzene ring is substituted with F, CN, Cl, SO_2_CH_3_, SC_6_H_5_, and NO_2_ (Figure 6(Fig. 6)). Among all, four thiophene-based modified compounds from series **2** exhibited the highest DPPH activity (%DPPH = 153.54-186.20). These top-four compounds are thiophene-containing compounds (**2d > 2a > 2t > 2v**: R = *para*-CN > *para*-F > *para*-Cl > *para*-SO_2_CH_3_). Additionally, *meta*-NO_2_ thiophene analog **2w** was also presented in the list of top-ten compounds with %DPPH = 126.76. Furan ring was shown to be an essential moiety for potent activity of the compounds from series **7** in which the compound **7t **bearing *para*-Cl (%DPPH = 133.58) was the most potent compound of the series, followed by the compound **7v **with *para*-SO_2_CH_3 _(%DPPH = 118.39). Additionally, two thiazole-based compounds **5i** (R = *para*-SC_6_H_5_, %DPPH = 133.03) and **11a** (R = naphthalene, %DPPH = 116.89) as well as the pyrrole-analog **3h **(R = *para*-Cl, %DPPH = 114.17) (Supplementary information, Table S6).

Seven out of ten most potent SOD-mimic agents are the members of modified compounds series **2** (Figure 7(Fig. 7)). Like the DPPH activity, these top-ranking compounds are thiophene-bearing analogs in which the *para*-position of their terminal benzene ring is substituted with F, CH_3_, CF_3_, OCH_3_, and COOCH_3_ as well as tetra-CH_3 _groups. Moreover, thiazole-bearing compounds **5a** (R = OC_6_H_5_) and **4c** (R = COOCH_3_) exhibited comparable activity with %SOD = 114.72 and 114.11, respectively. Moreover, a pyrrole-based compound **3o** (R = CF_3_, %SOD = 102.91) was included in the top ten list.

In overview, the introduction of the thiophene ring to the sulfonamide core and substitutions of electron withdrawing groups (i.e., CN, Cl and F) on terminal benzene ring of the parent compounds provided the most promising effects on improving antioxidant (both DPPH and SOD) activities of the compounds.

Antioxidant agents have been documented for their therapeutic applications as single or combined drugs for several diseases including cancers (Khurana et al., 2018[23]; Marino et al., 2023[30]; Neha et al., 2019[34]; Singh et al., 2018[46]). Cancerous cells display the unique characteristic of requiring the high reactive oxidative stress (ROS) condition to maintain their high rate of proliferation. The ability to neutralize the free radicals renders the use of antioxidants beneficial for decreasing cellular survival rate. The use of antioxidant agents for combined therapy with conventional chemotherapeutic drugs for enhancing potency as well as minimizing oxidative-related side effects and toxicities was also reported (Asnaashari et al., 2023[2]; Wongsawatkul et al., 2024[52]). Many ongoing clinical studies of using antioxidant agents for cancer therapeutics have been reported (Luo et al., 2022[28]). Several studies suggested the positive effects of using antioxidant supplements as conjunctives for chemotherapy. However, their enhancing impact on therapeutic outcomes is still controversial (Khurana et al., 2018[23]; Saeidnia and Abdollahi, 2013[44]; Singh et al., 2018[46]). Besides cancers, antioxidant agents have been recognized for their benefits on preventing or treating many oxidative-related diseases including cardiovascular and neurodegenerative diseases (Blagov et al., 2024[9]; Neha et al., 2019[34]; Zhang et al., 2015[57]).

## Conclusion

A series of 2-aminothiazole derivatives (**1-12**) were synthesized and investigated for their antioxidant and antimicrobial effects. From the antioxidant studies using DPPH and SOD assays, it was indicated that most of the compounds are active antioxidants, except for compound **1** (inactive SOD activity). The most potent antioxidant compounds were noted to be *para-*Cl compound **8** with DPPH IC_50 _= 109.73 µM and SOD IC_50 _= 188.27 µM. Unfortunately, all tested compounds displayed none of antimicrobial effects. Two antioxidant QSAR models (i.e., DPPH and SOD) were successfully constructed using MLR algorithm. Both constructed models displayed good predictive performance as indicated by high correlation coefficient (*R^2^_Tr_* and *Q^2^_LOO.CV_*) and low root mean square (*RMSE*) values. Key structural features influencing antioxidant activities were revealed including mass (H6m, RDF040m, and Mor31m), polarizability (HATS8p), electronegativity (Mor13e), as well as structural symmetry (Gu) and the presence of C-F bond (B01[C-F]). The models were further employed to guide the rational design and predict antioxidant activities of an additional set of 112 modified compounds. Most of the modified compounds demonstrated improved activities when compared to their parents, particularly, compound **2a** with promising DPPH and SOD predicted activities. Structure-activity relationship analysis suggested that the replacement of thiazole ring with the thiophene ring along with the substitution of electron-withdrawing group (i.e., CN, Cl, and F) at *para-*position of the terminal benzene ring could preferably increase antioxidant activities of the thiazole sulfonamides. The key findings obtained herein would be beneficial for the design, screening, and development of the related compounds as antioxidants for future therapeutic applications. 

The current study demonstrates the effective utilization of computational tools in facilitating the design of new potent antioxidants. However, the synthesis and further studies (*in vitro,* *in vivo, *and clinical trials*)* to validate the antioxidant effects of these newly designed compounds are recommended. Pharmacokinetics profiles and drug-likeness of the compounds are crucial factors contributing to clinical health-promoting effects of the administered antioxidants. The antioxidant agents, particularly the natural-derived ones, were noted for their undesirable bioavailability (Danyo and Ivantsova, 2025[14]). Accordingly, studies on pharmacokinetics and toxicity profiles of these thiazole sulfonamide-based antioxidants are necessary for successful development. The structure-activity relationships findings obtained from this study would partially be helpful for future structural optimization to improve their bioavailability, stability, as well as drug-like profiles. Additional drug delivery technologies may also be further investigated for effective delivery and sustaining optimal concentration at target tissues (Gao et al., 2023[21]; Li et al., 2019[25]; Marino et al., 2022[29]). However, further synthesis and studies on antioxidant effects of the highlighted compounds as well as their pharmacokinetic properties are highly recommended for successful therapeutic applications.

## Notes

Apilak Worachartcheewan and Ratchanok Pingaew (Department of Chemistry, Faculty of Science, Srinakharinwirot University, Bangkok 10110, Thailand; Phone: +66-2-649-5000 ext 18253, Fax: +662-260-0128, E-mail: ratchanok@g.swu.ac.th) contributed equally as corresponding author.

## Declaration

### Author contribution statement

Apilak Worachartcheewan: Conceptualization, methodology, investigation, validation, visualization, formal analysis, resources, writing-original draft, funding acquisition 

Ratchanok Pingaew: Conceptualization, methodology, investigation, validation, formal analysis, resources, writing-original draft

Veda Prachayasittikul: Conceptualization, methodology, validation, formal analysis, visualization, writing- review & editing, supervision

Setthawut Apiraksattayakul: Investigation, formal analysis

Supaluk Prachayasittikul: Conceptualization, writing- review & editing, supervision

Somsak Ruchirawat: Resources, supervision

Virapong Prachayasittikul: Conceptualization, supervision

### Supplementary information

Supplementary information is available on EXCLI Journal's website.

### Conflict of interest

The authors declare that they have no conflict of interest.

### Acknowledgments

This work (Grant No. RGNS 63-155) was supported by Office of the Permanent Secretary, Ministry of Higher Education, Science, Research and Innovation (OPS MHESI), Thailand Science Research and Innovation (TSRI) and Mahidol University, and by Mahidol University (Fundamental Funds: fiscal year 2024 by National Science Research and Innovation Fund (NSRF)).

## Supplementary Material

Supplementary information

## Figures and Tables

**Table 1:**
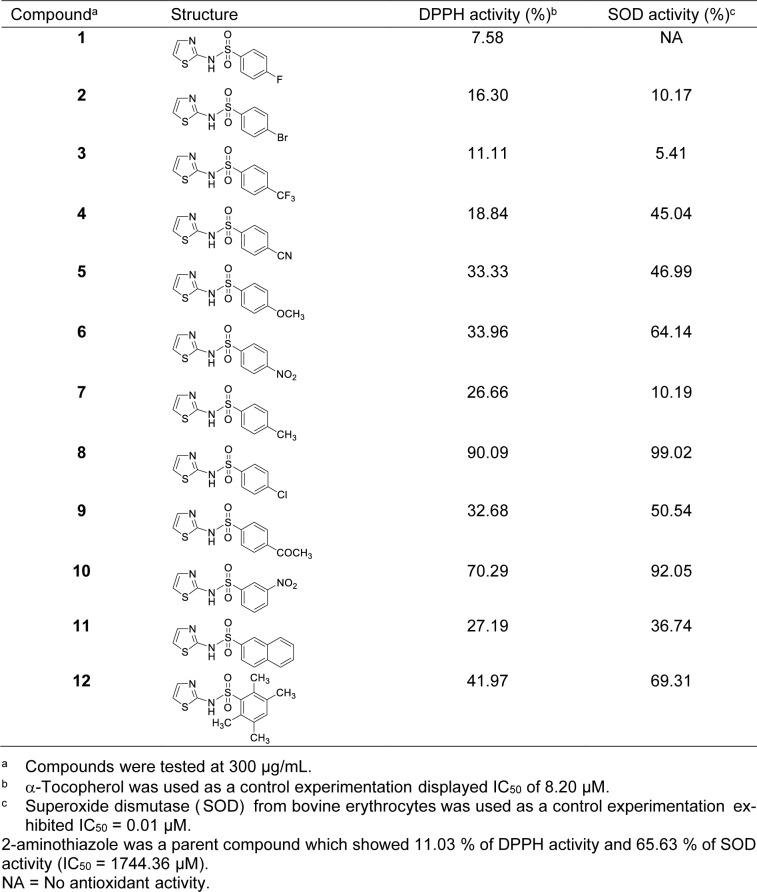
Antioxidant activity (%DPPH and %SOD) of 2-aminothiazole sulfonamide derivatives (1-12)

**Table 2:**
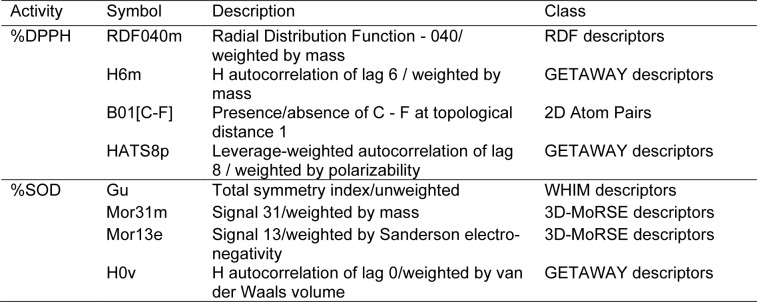
Definition and class of key descriptors for QSAR model construction

**Table 3:**
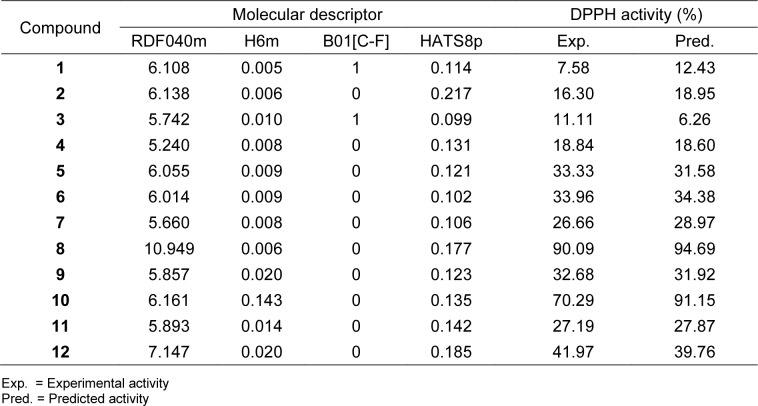
Values of key descriptors and experimental *vs* predicted antioxidant activity (%DPPH) of 2-aminothiazole sulfonamide derivatives

**Table 4:**
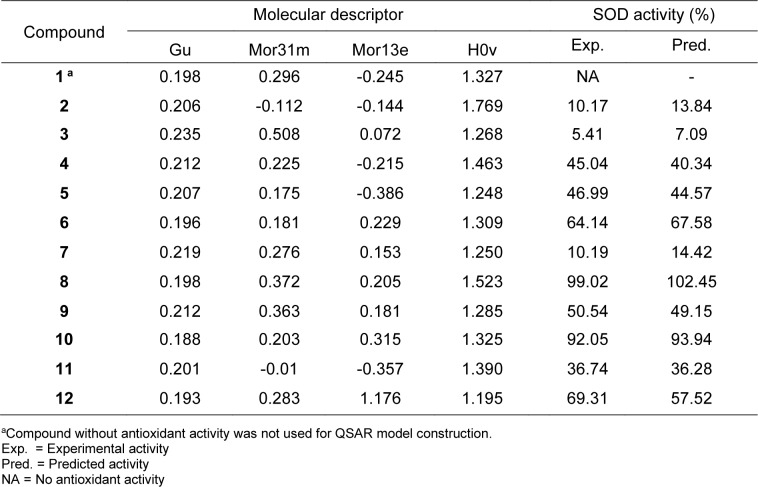
Values of key descriptors and experimental *vs* predicted antioxidant activity (%SOD) of 2-aminothiazole sulfonamide derivatives

**Table 5:**
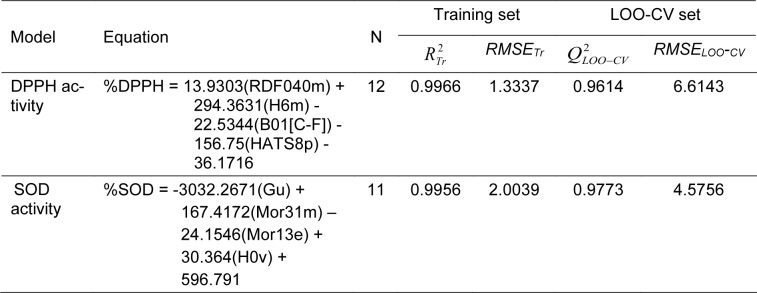
QSAR equations and summarized predictive performance

**Figure 1:**
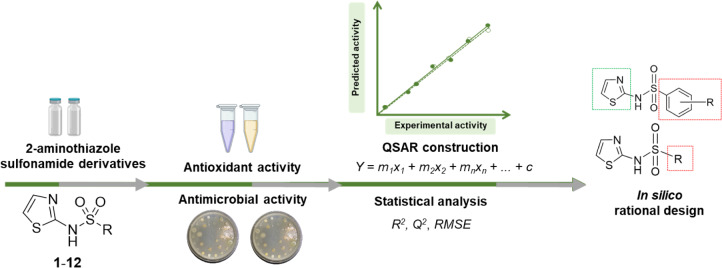
Graphical abstract
