# Supplementary material for: Synthesis, biological investigation, and in silico studies of 2-aminothiazole sulfonamide derivatives as potential antioxidants
Source: EXCLI J. 2025 Jan 3;24:60–81. doi: 10.17179/excli2024-7855 (PMC11830920; doi:10.17179/excli2024-7855)
Supplement: Supplementary information [file EXCLI-24-60-s-001.pdf]

EXCLI Journal 2025;24:60-81 – ISSN 1611-2156
Received: September 19, 2024, accepted: December 18, 2024, published: January 03, 2025

Original article:

SYNTHESIS, BIOLOGICAL INVESTIGATION, AND *IN SILICO* STUDIES OF 2-AMINOTHIAZOLE SULFONAMIDE DERIVATIVES AS POTENTIAL ANTIOXIDANTS

Apilak Worachartcheewan[1*], Ratchanok Pingaew[2*], Veda Prachayasittikul[3], Setthawut Apiraksattayakul[1], Supaluk Prachayasittikul[3], Somsak Ruchirawat[4,5,6], Virapong Prachayasittikul[7]

[1] Department of Community Medical Technology, Faculty of Medical Technology, Mahidol University, Bangkok 10700, Thailand
[2] Department of Chemistry, Faculty of Science, Srinakharinwirot University, Bangkok 10110, Thailand
[3] Center for Research Innovation and Biomedical Informatics, Faculty of Medical Technology, Mahidol University, Bangkok 10700, Thailand
[4] Laboratory of Medicinal Chemistry, Chulabhorn Research Institute, Bangkok 10210, Thailand
[5] Program in Chemical Sciences, Chulabhorn Graduate Institute, Bangkok 10210, Thailand
[6] Center of Excellence on Environmental Health and Toxicology (EHT), Commission on Higher Education, Ministry of Education, Bangkok 10400, Thailand
[7] Department of Clinical Microbiology and Applied Technology, Faculty of Medical Technology, Mahidol University, Bangkok 10700, Thailand

* **Corresponding authors:** Assistant Professor Dr. Apilak Worachartcheewan, Department of Community Medical Technology, Faculty of Medical Technology, Mahidol University, Bangkok 10700, Thailand, Phone: +66-2-441-4371-8 ext 2720; Fax: +662-441-4380; E-mail: apilak.woa@mahidol.ac.th
Associate Professor Dr. Ratchanok Pingaew, Department of Chemistry, Faculty of Science, Srinakharinwirot University, Bangkok 10110, Thailand,
Phone: +66-2-649-5000 ext 18253; Fax: +662-260-0128; E-mail: ratchanok@g.swu.ac.th

https://dx.doi.org/10.17179/excli2024-7855

This is an Open Access article distributed under the terms of the Creative Commons Attribution License (http://creativecommons.org/licenses/by/4.0/).

EXCLI Journal 2025;24:60-81 – ISSN 1611-2156
Received: September 19, 2024, accepted: December 18, 2024, published: January 03, 2025

Supplementary information contents *Page*

Figure S1: Structurally modified compounds using compounds **1**, **9** and **10** as prototypes S3
Figure S2: Structurally modified compounds using compound **2** as a prototype S3
Figure S3: Structurally modified compounds using compound **3** as a prototype S4
Figure S4: Structurally modified compounds using compound **4** as a prototype S4
Figure S5: Structurally modified compounds using compound **5** as a prototype S5
Figure S6: Structurally modified compounds using compound **7** as a prototype S5
Figure S7: Structurally modified compounds using compound **11** as a prototype S6
Table S1: Intercorrelation matrix of molecular descriptors for DPPH model..................................... S7
Table S2: Intercorrelation matrix of molecular descriptors for SOD model....................................... S7
Table S3: Summary of predicted antioxidant activities of 112 modified compounds........................ S8
Table S4: Predicted DPPH (%) activity and descriptor values of 112 modified compounds S10
Table S5: Predicted SOD (%) activity and descriptor values of 112 modified compounds S13
Table S6: Top ten modified compounds with the highest DPPH and SOD activities (%)............... S16

EXCLI Journal 2025;24:60-81 – ISSN 1611-2156
Received: September 19, 2024, accepted: December 18, 2024, published: January 03, 2025

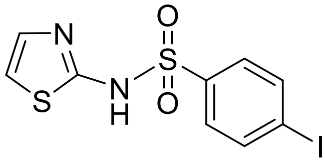

1a

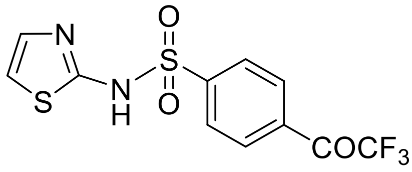

9a

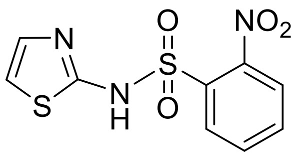

10a

Figure S1: Structurally modified compounds using compounds **1**, **9** and **10** as prototypes

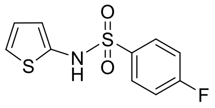

2a

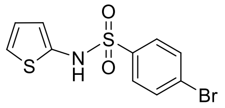

2b

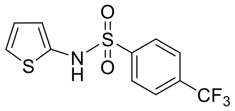

2c

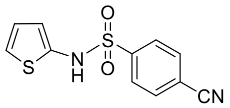

2d

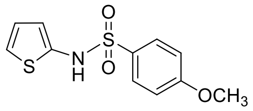

2e

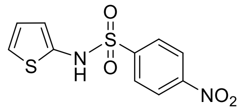

2f

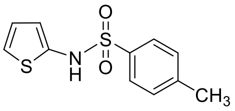

2g

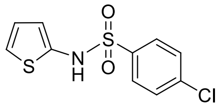

2h

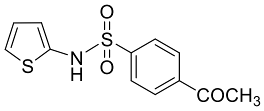

2i

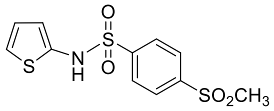

2j

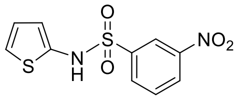

2k

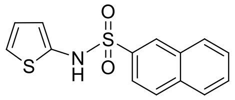

2l

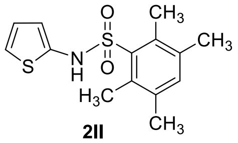

2ll

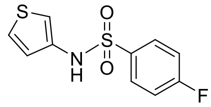

2m

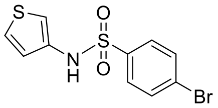

2n

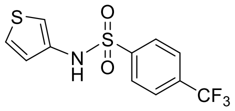

2o

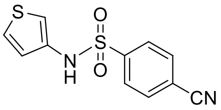

2p

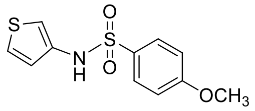

2q

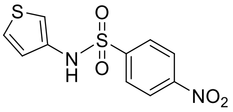

2r

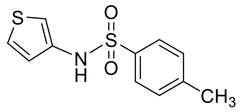

2s

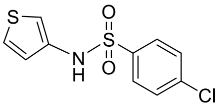

2t

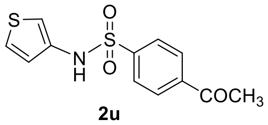

2u

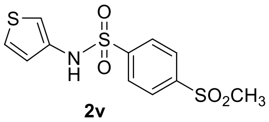

2v

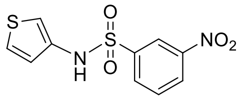

2w

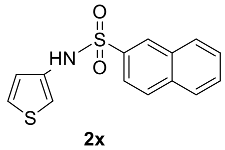

2x

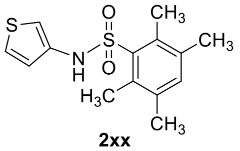

2xx

Figure S2: Structurally modified compounds using compound **2** as a prototype

EXCLI Journal 2025;24:60-81 – ISSN 1611-2156
Received: September 19, 2024, accepted: December 18, 2024, published: January 03, 2025

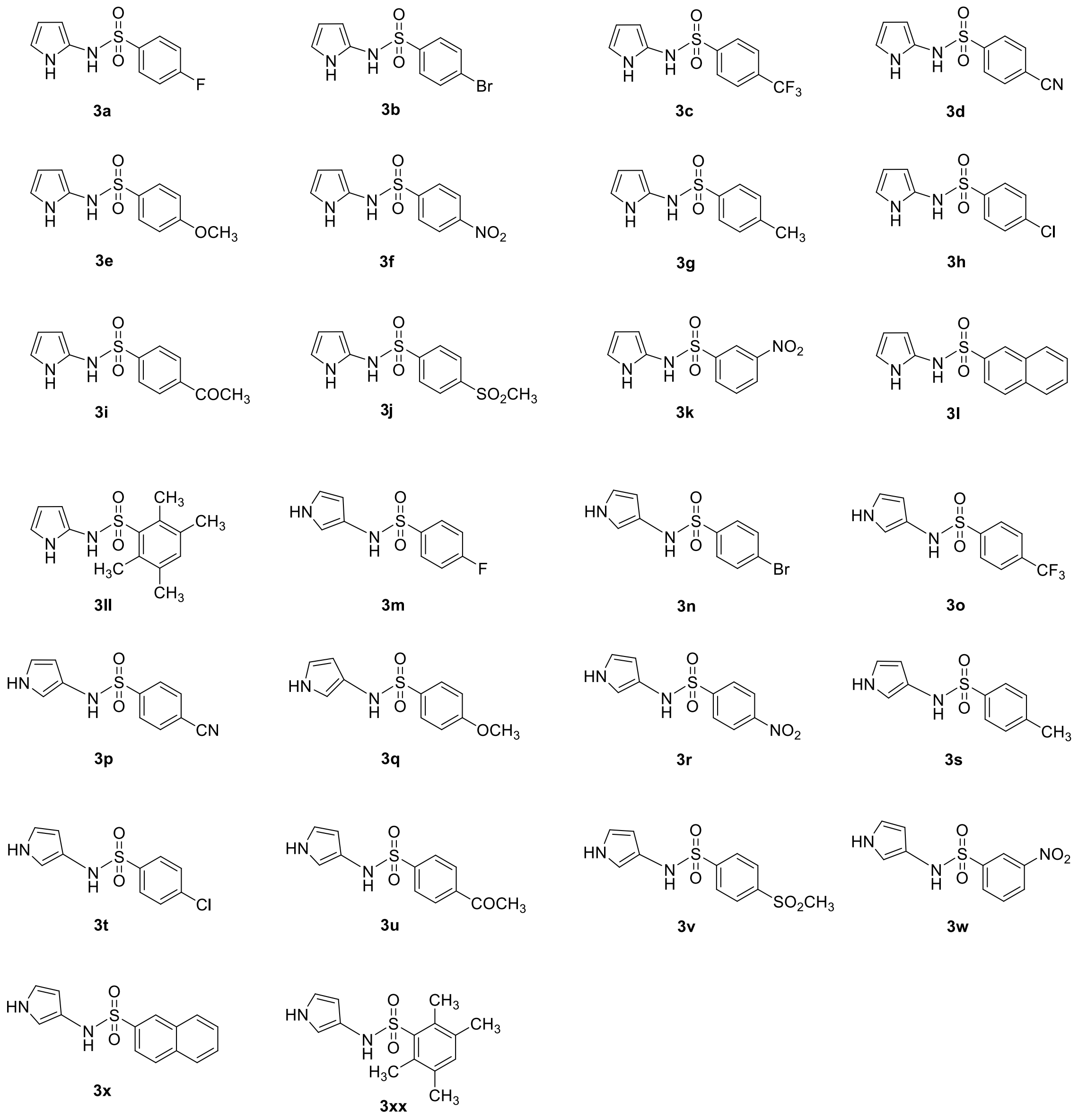

Figure S3: Structurally modified compounds using compound **3** as a prototype

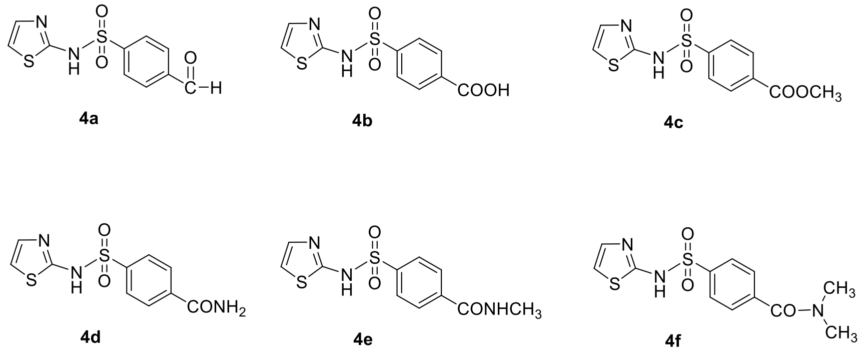

Figure S4: Structurally modified compounds using compound **4** as a prototype

EXCLI Journal 2025;24:60-81 – ISSN 1611-2156
Received: September 19, 2024, accepted: December 18, 2024, published: January 03, 2025

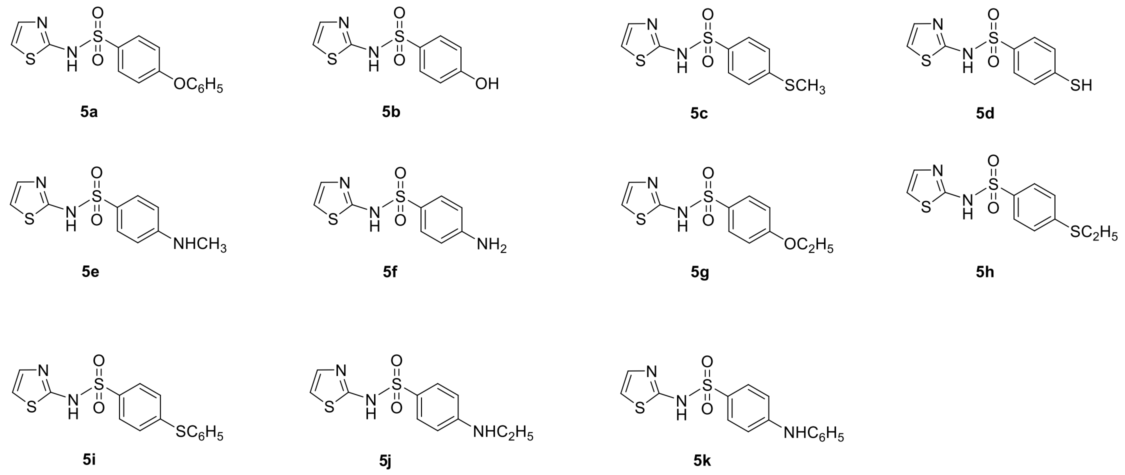

Figure S5: Structurally modified compounds using compound **5** as a prototype

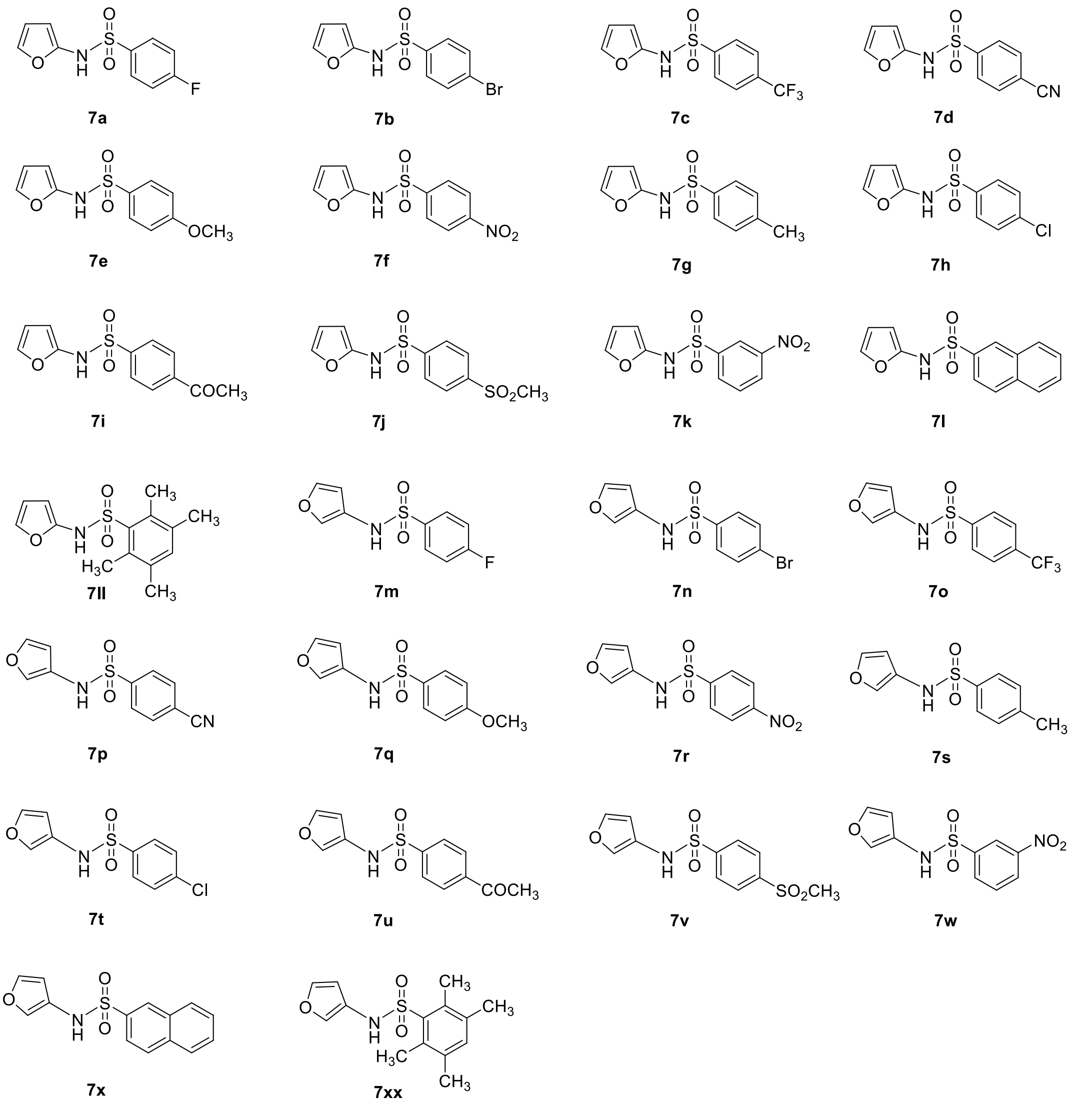

Figure S6: Structurally modified compounds using compound **7** as a prototype

EXCLI Journal 2025;24:60-81 – ISSN 1611-2156
Received: September 19, 2024, accepted: December 18, 2024, published: January 03, 2025

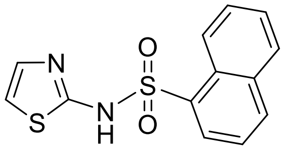

11a

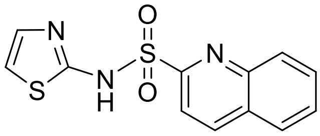

11b

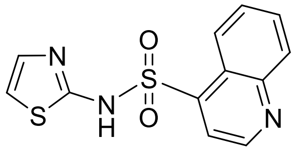

11c

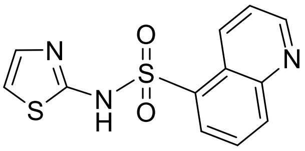

11d

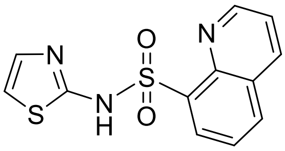

11e

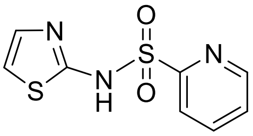

11f

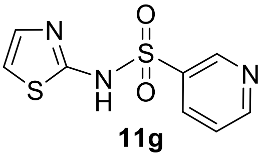

11g

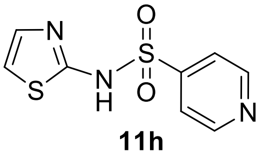

11h

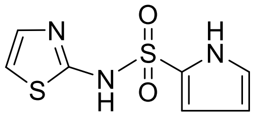

11i

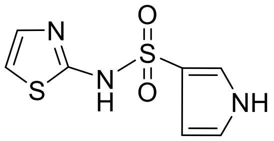

11j

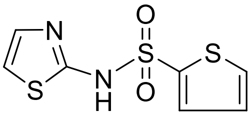

11k

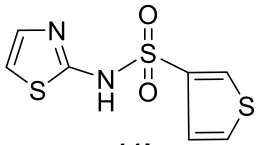

11l

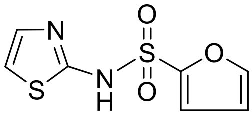

11m

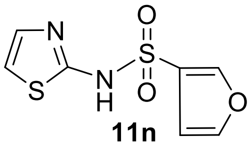

11n

Figure S7: Structurally modified compounds using compound **11** as a prototype

EXCLI Journal 2025;24:60-81 – ISSN 1611-2156
Received: September 19, 2024, accepted: December 18, 2024, published: January 03, 2025

Table S1: Intercorrelation matrix of molecular descriptors for DPPH model

	RDF040m	H6m	B01[C-F]	HATS8p
RDF040m	1.000			
H6m	-0.071	1.000		
B01[C-F]	-0.153	-0.169	1.000	
HATS8p	0.466	-0.015	-0.395	1.000

Table S2: Intercorrelation matrix of molecular descriptors for SOD model

	Gu	Mor31m	Mor13e	H0v
Gu	1.000			
Mor31m	0.423	1.000		
Mor13e	-0.346	0.386	1.000	
H0v	-0.101	-0.570	-0.388	1.000

EXCLI Journal 2025;24:60-81 – ISSN 1611-2156
Received: September 19, 2024, accepted: December 18, 2024, published: January 03, 2025

Table S3: Summary of predicted antioxidant activities of 112 modified compounds

Compound prototype	Antioxidant activity		Number of modified compound	DPPH (%) activity, *N*			SOD (%) activity, *N*		
	DPPH (%)	SOD (%)		Improved	Decreased	NA	Improved	Decreased	NA
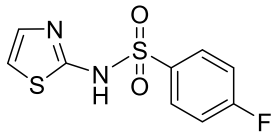 **1**	7.58	NA	1	0	0	1	0	0	1
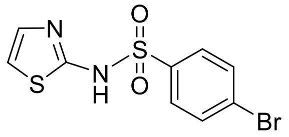 **2**	16.30	10.17	26	25	1	0	24	2	0
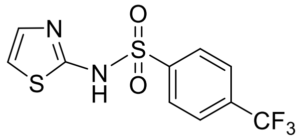 **3**	11.11	5.41	26	26	0	0	15	4	7
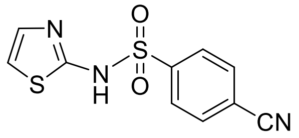 **4**	18.84	45.04	6	6	0	0	2	3	1
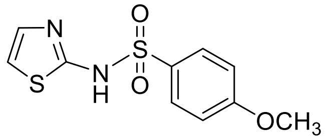 **5**	33.33	46.99	11	8	3	0	10	1	0

Compound prototype	Antioxidant activity		Number of modified compound	DPPH (%) activity, *N*			SOD (%) activity, *N*		
	DPPH (%)	SOD (%)		Improved	Decreased	NA	Improved	Decreased	NA
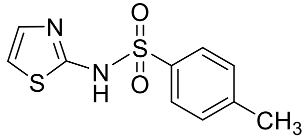 **7**	33.96	64.14	26	19	7	0	3	15	8
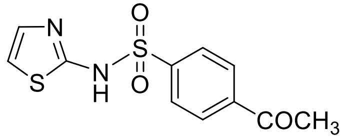 **9**	26.66	10.19	1	0	1	0	1	0	0
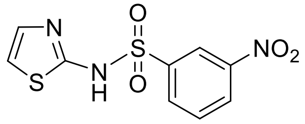 **10**	70.29	92.05	1	0	1	0	0	0	1
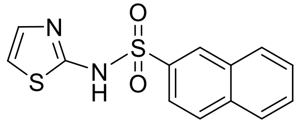 **11**	27.19	36.74	14	4	8	2	9	3	2

Improved activity: the value of predicted activity (%) is greater than antioxidant activity that of the parent compound.
Decreased activity: the value of predicted activity (%) is between 0 and antioxidant activity that of the parent compound.
No antioxidant activity: the value of predicted activity (%) is less than 0.
NA = No antioxidant activity.

EXCLI Journal 2025;24:60-81 – ISSN 1611-2156
Received: September 19, 2024, accepted: December 18, 2024, published: January 03, 2025

Table S4: Predicted DPPH (%) activity and descriptor values of 112 modified compounds

No.	Code	Descriptors				Predicted DPPH (%)[a]
		RDF040m	H6m	B01[C-F]	HATS8p	
1	1a	4.846	0.005	0	0.318	-17.04
2	2a	17.805	0.037	1	0.133	179.37
3	2b	6.234	0.016	0	0.216	21.52
4	2c	5.869	0.017	1	0.106	11.44
5	2d	17.123	0.025	0	0.15	186.20
6	2e	6.176	0.039	0	0.117	43.00
7	2f	6.064	0.016	0	0.111	35.61
8	2g	5.961	0.018	0	0.111	34.77
9	2h	11.065	0.016	0	0.183	93.99
10	2i	5.896	0.037	0	0.117	38.51
11	2j	9.523	0.046	0	0.121	91.06
12	2k	6.414	0.095	0	0.142	58.88
13	2l	5.716	0.037	0	0.135	33.18
14	2ll	10.05	0.032	0	0.184	84.41
15	2m	11.081	0.006	1	0.169	70.93
16	2n	11.281	0.006	0	0.233	86.22
17	2o	10.855	0.009	1	0.118	76.66
18	2p	10.349	0.008	0	0.161	85.11
19	2q	10.772	0.01	0	0.127	96.92
20	2r	10.988	0.008	0	0.13	98.87
21	2s	10.556	0.008	0	0.124	93.79
22	2t	16.071	0.006	0	0.208	156.86
23	2u	10.838	0.01	0	0.123	98.47
24	2v	14.538	0.022	0	0.123	153.54
25	2w	11.237	0.116	0	0.177	126.76
26	2x	10.796	0.026	0	0.155	97.58
27	2xx	11.848	0.022	0	0.227	99.77
28	3a	7.467	0.01	1	0.125	28.66
29	3b	7.436	0.01	0	0.167	44.18
30	3c	7.117	0.015	1	0.096	29.80
31	3d	6.406	0.011	0	0.124	36.87
32	3e	7.877	0.021	0	0.11	62.50
33	3f	7.216	0.012	0	0.104	51.58
34	3g	7.509	0.015	0	0.1	57.17
35	3h	12.269	0.01	0	0.15	114.17
36	3i	7.341	0.014	0	0.107	53.44
37	3j	11.019	0.015	0	0.113	104.03
38	3k	7.281	0.07	0	0.126	66.11
39	3l	7.867	0.032	0	0.119	64.18
40	3ll	8.719	0.006	0	0.165	61.19
41	3m	7.129	0.007	1	0.117	24.32
42	3n	6.215	0.007	0	0.168	26.13
43	3o	5.905	0.009	1	0.093	11.62

EXCLI Journal 2025;24:60-81 – ISSN 1611-2156
Received: September 19, 2024, accepted: December 18, 2024, published: January 03, 2025

No.	Code	Descriptors				Predicted DPPH (%)[a]
		RDF040m	H6m	B01[C-F]	HATS8p	
**44**	**3p**	5.507	0.008	0	0.121	23.93
**45**	**3q**	6.215	0.013	0	0.107	37.46
**46**	**3r**	6.2	0.009	0	0.1	37.17
**47**	**3s**	6.162	0.009	0	0.097	37.11
**48**	**3t**	11.139	0.007	0	0.149	97.70
**49**	**3u**	6.084	0.012	0	0.106	35.50
**50**	**3v**	9.511	0.021	0	0.105	86.04
**51**	**3w**	7.141	0.066	0	0.125	63.14
**52**	**3x**	6.327	0.023	0	0.118	40.24
**53**	**3xx**	8.714	0.007	0	0.153	63.29
**54**	**4a**	5.529	0.01	0	0.114	25.92
**55**	**4b**	5.488	0.005	0	0.116	23.57
**56**	**4c**	6.19	0.011	0	0.127	33.39
**57**	**4d**	5.767	0.02	0	0.116	31.87
**58**	**4e**	5.661	0.097	0	0.121	52.27
**59**	**4f**	5.918	0.018	0	0.119	32.91
**60**	**5a**	6.112	0.06	0	0.124	47.20
**61**	**5b**	5.818	0.006	0	0.108	29.71
**62**	**5c**	9.1	0.008	0	0.156	68.50
**63**	**5d**	9.018	0.006	0	0.168	64.88
**64**	**5e**	6.119	0.008	0	0.117	33.08
**65**	**5f**	5.962	0.007	0	0.105	32.48
**66**	**5g**	6.26	0.026	0	0.116	40.50
**67**	**5h**	9.131	0.029	0	0.136	78.24
**68**	**5i**	12.227	0.075	0	0.148	133.03
**69**	**5j**	6.183	0.023	0	0.113	39.02
**70**	**5k**	6.235	0.029	0	0.122	40.10
**71**	**7a**	5.711	0.005	1	0.123	3.04
**72**	**7b**	5.794	0.005	0	0.158	21.25
**73**	**7c**	5.422	0.007	1	0.097	3.68
**74**	**7d**	5.022	0.006	0	0.123	16.27
**75**	**7e**	6.848	0.001	0	0.117	41.18
**76**	**7f**	5.846	0.006	0	0.106	30.42
**77**	**7g**	6.411	0.001	0	0.1	37.75
**78**	**7h**	10.63	0.005	0	0.144	90.81
**79**	**7i**	5.417	0.009	0	0.112	24.38
**80**	**7j**	9.446	0.021	0	0.109	84.51
**81**	**7k**	6.005	0.112	0	0.119	61.80
**82**	**7l**	5.505	0.017	0	0.116	27.34
**83**	**7ll**	8.309	0.004	0	0.164	55.05
**84**	**7m**	8.802	0.005	1	0.11	48.14
**85**	**7n**	8.919	0.005	0	0.175	62.11
**86**	**7o**	8.603	0.007	1	0.088	49.40
**87**	**7p**	8.052	0.006	0	0.117	59.42

EXCLI Journal 2025;24:60-81 – ISSN 1611-2156
Received: September 19, 2024, accepted: December 18, 2024, published: January 03, 2025

No.	Code	Descriptors				Predicted DPPH (%)[a]
		RDF040m	H6m	B01[C-F]	HATS8p	
**88**	**7q**	8.618	0.012	0	0.101	71.58
**89**	**7r**	8.858	0.007	0	0.094	74.55
**90**	**7s**	8.407	0.007	0	0.092	68.58
**91**	**7t**	13.768	0.005	0	0.15	133.58
**92**	**7u**	8.716	0.012	0	0.101	72.95
**93**	**7v**	12.153	0.008	0	0.109	118.39
**94**	**7w**	8.656	0.111	0	0.121	98.12
**95**	**7x**	7.485	0.011	0	0.12	52.52
**96**	**7xx**	8.912	0.003	0	0.164	63.15
**97**	**9a**	5.772	0.031	1	0.117	12.49
**98**	**10a**	5.785	0.056	0	0.149	37.54
**99**	**11a**	11.114	0.084	0	0.169	116.89
**100**	**11b**	5.155	0.012	0	0.147	16.13
**101**	**11c**	5.218	0.032	0	0.163	20.39
**102**	**11d**	10.245	0.087	0	0.159	107.23
**103**	**11e**	6.153	0.021	0	0.164	30.02
**104**	**11f**	4.042	0.002	0	0.136	-0.59
**105**	**11g**	6.32	0.012	0	0.114	37.53
**106**	**11h**	5.295	0.006	0	0.098	23.99
**107**	**11i**	3.99	0.016	0	0.074	12.52
**108**	**11j**	4.4	0.012	0	0.076	16.74
**109**	**11k**	3.952	0.003	0	0.093	5.19
**110**	**11l**	4.125	0.008	0	0.104	7.34
**111**	**11m**	3.204	0.001	0	0.083	-4.25
**112**	**11n**	4.682	0.009	0	0.067	21.20

[a]Predicted activity was calculated using the following equation:
%DPPH = 13.9303(RDF040m) + 294.3631(H6m) - 22.5344(B01[C-F]) - 156.75(HATS8p) - 36.1716

EXCLI Journal 2025;24:60-81 – ISSN 1611-2156
Received: September 19, 2024, accepted: December 18, 2024, published: January 03, 2025

Table S5: Predicted SOD (%) activity and descriptor values of 112 modified compounds

No.	Code	Descriptors				Predicted SOD
		Gu	Mor31m	Mor13e	H0v	
1	1a	0.232	-0.254	0.112	2.072	-89.01
2	2a	0.191	0.332	-0.458	1.318	124.29
3	2b	0.198	-0.19	-0.031	1.704	17.08
4	2c	0.194	0.332	0.119	1.245	99.04
5	2d	0.2	0.185	-0.441	1.444	75.81
6	2e	0.179	0.095	0.064	1.211	105.14
7	2f	0.183	-0.025	0.171	1.265	71.98
8	2g	0.212	0.169	0.396	1.224	9.84
9	2h	0.198	0.218	0.25	1.475	71.65
10	2i	0.196	0.256	0.153	1.249	79.55
11	2j	0.2	0.125	0.059	1.242	47.55
12	2k	0.189	0.009	0.364	1.286	55.46
13	2l	0.193	-0.079	0.043	1.345	38.14
14	2ll	0.176	0.156	0.802	1.184	105.81
15	2m	0.203	0.175	-0.439	1.378	62.98
16	2n	0.195	-0.157	-0.418	1.795	43.81
17	2o	0.194	0.351	-0.23	1.33	113.23
18	2p	0.2	0.024	-0.386	1.505	49.38
19	2q	0.196	0.14	-0.466	1.312	77.00
20	2r	0.193	-0.048	0.033	1.354	43.84
21	2s	0.181	0.159	-0.074	1.312	116.19
22	2t	0.203	0.225	-0.071	1.563	68.08
23	2u	0.183	0.199	-0.238	1.348	121.88
24	2v	0.205	0.105	-0.196	1.313	37.36
25	2w	0.21	0.036	-0.012	1.38	8.23
26	2x	0.193	-0.112	-0.238	1.439	42.26
27	2xx	0.167	-0.046	0.506	1.231	107.86
28	3a	0.205	-0.064	-0.085	1.164	1.86
29	3b	0.21	-0.282	-0.041	1.567	-38.63
30	3c	0.19	0.089	0.237	1.129	64.12
31	3d	0.197	-0.231	-0.091	1.291	2.16
32	3e	0.183	-0.131	0.039	1.105	52.56
33	3f	0.2	-0.295	0.222	1.146	-29.62
34	3g	0.185	-0.041	0.293	1.105	55.43
35	3h	0.185	-0.051	0.224	1.343	62.65
36	3i	0.21	-0.002	0.201	1.143	-10.47
37	3j	0.211	-0.159	-0.024	1.162	-33.77
38	3k	0.174	-0.265	0.41	1.17	50.43
39	3l	0.179	-0.34	-0.178	1.235	38.89
40	3ll	0.173	-0.139	1.177	1.105	54.06
41	3m	0.177	-0.063	-0.18	1.141	88.53
42	3n	0.191	-0.265	-0.152	1.556	24.18
43	3o	0.182	0.141	-0.022	1.115	102.91

No.	Code	Descriptors				Predicted SOD
		Gu	Mor31m	Mor13e	H0v	
**44**	**3p**	0.189	-0.202	-0.22	1.28	34.05
**45**	**3q**	0.18	-0.075	0.011	1.092	71.32
**46**	**3r**	0.201	-0.244	0.065	1.134	-20.68
**47**	**3s**	0.193	-0.007	0.335	1.093	35.49
**48**	**3t**	0.185	-0.005	0.141	1.333	72.05
**49**	**3u**	0.208	0.014	-0.073	1.132	4.56
**50**	**3v**	0.194	-0.099	0.005	1.112	25.60
**51**	**3w**	0.215	-0.25	0.186	1.162	-66.21
**52**	**3x**	0.194	-0.322	-0.378	1.229	1.07
**53**	**3xx**	0.195	-0.146	1.246	1.079	-16.28
**54**	**4a**	0.23	0.23	0.135	1.341	-24.67
**55**	**4b**	0.218	0.227	0.031	1.304	12.61
**56**	**4c**	0.183	0.215	0.104	1.276	114.11
**57**	**4d**	0.215	0.225	-0.215	1.293	26.98
**58**	**4e**	0.211	0.225	0.352	1.206	22.77
**59**	**4f**	0.195	0.29	0.361	1.206	81.95
**60**	**5a**	0.184	0.133	-0.503	1.365	114.72
**61**	**5b**	0.195	0.222	-0.464	1.272	92.50
**62**	**5c**	0.184	0.152	0.148	1.352	101.78
**63**	**5d**	0.22	0.361	0.058	1.428	32.09
**64**	**5e**	0.186	0.09	-0.394	1.231	94.75
**65**	**5f**	0.191	0.071	-0.455	1.254	78.58
**66**	**5g**	0.193	0.211	-0.199	1.236	89.23
**67**	**5h**	0.201	0.132	0.002	1.3	48.83
**68**	**5i**	0.188	0.162	0.062	1.443	96.16
**69**	**5j**	0.196	0.078	-0.295	1.22	59.69
**70**	**5k**	0.18	-0.012	-0.373	1.378	99.83
**71**	**7a**	0.199	0.036	-0.043	1.181	36.30
**72**	**7b**	0.203	-0.275	0.076	1.583	-18.57
**73**	**7c**	0.188	0.18	0.184	1.145	87.18
**74**	**7d**	0.223	-0.122	-0.057	1.308	-58.74
**75**	**7e**	0.192	-0.146	0.073	1.126	22.58
**76**	**7f**	0.226	-0.192	0.279	1.164	-92.04
**77**	**7g**	0.202	-0.064	0.399	1.122	-2.01
**78**	**7h**	0.203	0.059	0.402	1.36	22.70
**79**	**7i**	0.183	0.098	0.299	1.162	86.35
**80**	**7j**	0.198	-0.06	0.218	1.142	15.77
**81**	**7k**	0.205	-0.145	0.604	1.193	-27.46
**82**	**7l**	0.182	-0.239	-0.268	1.257	49.55
**83**	**7ll**	0.198	-0.005	1.162	1.107	1.11
**84**	**7m**	0.187	0.069	-0.25	1.181	83.21
**85**	**7n**	0.21	-0.179	-0.19	1.6	-16.78
**86**	**7o**	0.228	0.172	-0.082	1.142	-29.11
**87**	**7p**	0.196	-0.17	-0.231	1.31	19.36

EXCLI Journal 2025;24:60-81 – ISSN 1611-2156
Received: September 19, 2024, accepted: December 18, 2024, published: January 03, 2025

No.	Code	Descriptors				Predicted SOD
		Gu	Mor31m	Mor13e	H0v	
**88**	**7q**	0.198	0.003	-0.23	1.117	36.38
**89**	**7r**	0.212	-0.229	0.074	1.16	-50.95
**90**	**7s**	0.194	0.036	0.144	1.119	45.06
**91**	**7t**	0.203	-0.014	0.169	1.367	16.32
**92**	**7u**	0.202	0.054	-0.026	1.154	28.98
**93**	**7v**	0.187	-0.067	-0.243	1.17	59.94
**94**	**7w**	0.197	-0.095	0.231	1.185	13.93
**95**	**7x**	0.198	-0.243	-0.413	1.268	4.20
**96**	**7xx**	0.181	-0.049	1.206	1.112	44.38
**97**	**9a**	0.215	0.524	0.378	1.304	63.04
**98**	**10a**	0.203	-0.158	-0.049	1.324	-3.83
**99**	**11a**	0.215	0.061	-0.19	1.28	-1.48
**100**	**11b**	0.185	-0.052	-0.258	1.391	75.58
**101**	**11c**	0.202	0.187	-0.335	1.374	65.39
**102**	**11d**	0.207	0.186	-0.196	1.276	43.73
**103**	**11e**	0.179	-0.024	0.103	1.383	89.50
**104**	**11f**	0.218	0.051	0.314	1.299	-23.85
**105**	**11g**	0.2	0.309	-0.024	1.324	82.85
**106**	**11h**	0.2	0.409	0.263	1.336	93.02
**107**	**11i**	0.203	0.247	0.774	1.285	42.91
**108**	**11j**	0.212	0.217	0.601	1.252	13.78
**109**	**11k**	0.198	0.374	0.022	1.421	101.63
**110**	**11l**	0.218	0.678	0.112	1.468	91.13
**111**	**11m**	0.204	0.16	0.683	1.271	27.09
**112**	**11n**	0.208	0.162	0.624	1.283	17.09

[a] Predicted activity was calculated using the following equation:
%SOD = -3032.2671(Gu) + 167.4172(Mor31m) - 24.1546(Mor13e) + 30.364(H0v) + 596.791

EXCLI Journal 2025;24:60-81 – ISSN 1611-2156
Received: September 19, 2024, accepted: December 18, 2024, published: January 03, 2025

Table S6: Top ten modified compounds with the highest DPPH and SOD activities (%)

Compound	DPPH activity (%)[a]	Compound	SOD activity (%)[a]
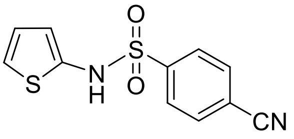 **2d**	186.20	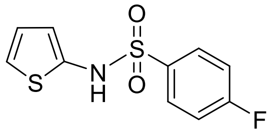 **2a**	124.29
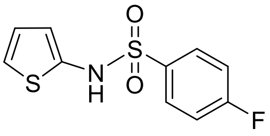 **2a**	179.37	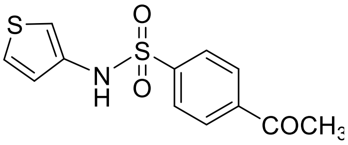 **2u**	121.88
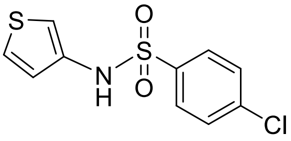 **2t**	156.86	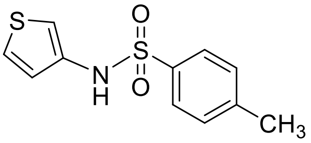 **2s**	116.19
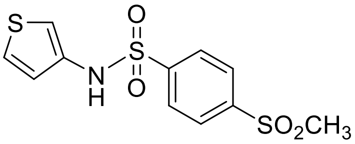 **2v**	153.54	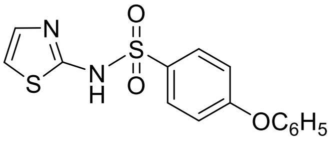 **5a**	114.72
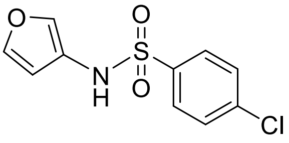 **7t**	133.58	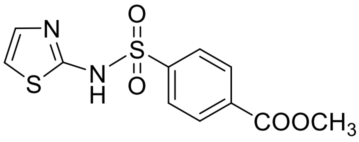 **4c**	114.11
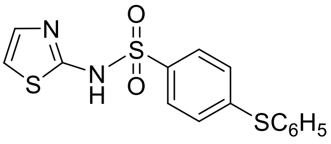 **5i**	133.03	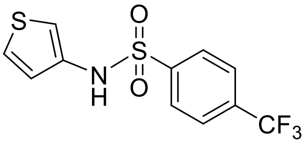 **2o**	113.23
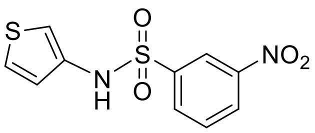 **2w**	126.76	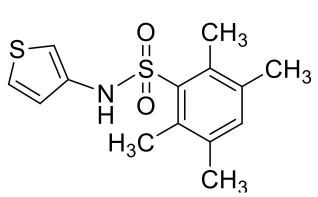 **2xx**	107.86

EXCLI Journal 2025;24:60-81 – ISSN 1611-2156
Received: September 19, 2024, accepted: December 18, 2024, published: January 03, 2025

Compound	DPPH activity (%)[a]	Compound	SOD activity (%)[a]
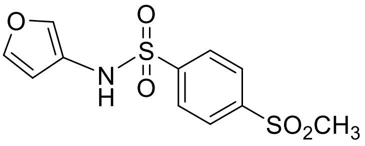 **7v**	118.39	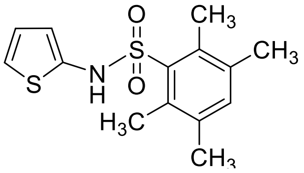 **2ll**	105.81
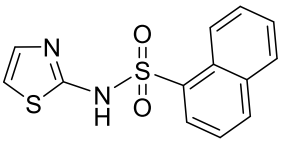 **11a**	116.89	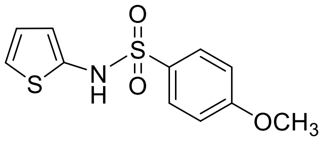 **2e**	105.14
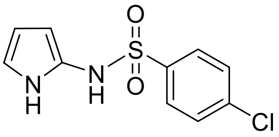 **3h**	114.17	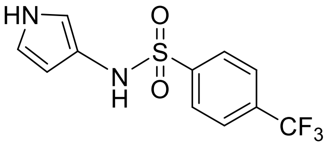 **3o**	102.91

[a]All top ten compounds displayed higher predicted activity when compared to their parent compounds.
